# Evaluating the prognostic value of body mass index in diffuse large B-cell lymphoma: a systematic review and meta-analysis

**DOI:** 10.3389/fnut.2026.1762123

**Published:** 2026-06-23

**Authors:** Liang Su, Yingjie Tian, Yujin Li, Yuchu Zhang, An Chang, Guoxing Yuan, Yongzheng Jiao, Jie Wu

**Affiliations:** 1 Guang'anmen Hospital, China Academy of Chinese Medical Sciences, Beijing, China; 2 Beijing University of Chinese Medicine, Beijing, China; 3 The First People's Hospital of Yunnan Province, The Affiliated Hospital of Kunming University of Science and Technology, Kunming, Yunnan, China

**Keywords:** body mass index, diffuse large B-cell lymphoma, obesity, overall survival, progression-free survival

## Abstract

**Background:**

The prognostic significance of body mass index (BMI) in diffuse large B-cell lymphoma (DLBCL) remains uncertain. We conducted a systematic review and meta-analysis to evaluate the associations between BMI and overall survival (OS) and progression-free survival (PFS) in patients with DLBCL.

**Methods:**

A systematic search of the Cochrane Library, PubMed, and Embase was conducted up to April 14, 2026, with additional reference screening. Random-effects models were used to calculate pooled hazard ratios (HRs) and 95% confidence intervals (CIs).

**Results:**

Of 764 records identified, 23 studies were included. In the main analyses, overweight status, defined according to study-specific thresholds, including Asian-specific cutoffs where applicable, was associated with better OS (HR = 0.82, 95% CI: 0.73–0.92, *P* = 0.001) and PFS (HR = 0.83, 95% CI: 0.72–0.96, *P* = 0.011). These associations remained consistent in subgroup analyses restricted to studies using R-CHOP-like regimens, multivariable-adjusted estimates, and normal-weight patients as the reference group. In BMI category-specific analyses, underweight status was associated with inferior OS (HR = 1.85, 95% CI: 1.30–2.64, *P* = 0.001) and PFS (HR = 1.64, 95% CI: 1.11–2.42, *P* = 0.013). By contrast, overweight status was associated with better OS (HR = 0.77, 95% CI: 0.70–0.84, *P* < 0.001) and PFS (HR = 0.80, 95% CI: 0.69–0.93, *P* = 0.004). Obesity was not significantly associated with either OS (HR = 0.93, 95% CI: 0.75–1.15, *P* = 0.508) or PFS (HR = 0.95, 95% CI: 0.80–1.13, *P* = 0.541).

**Conclusion:**

Available evidence suggests that overweight status may be associated with better survival outcomes in DLBCL, whereas underweight status may be associated with poorer prognosis. Current evidence remains insufficient to confirm a significant prognostic impact of obesity.

**Systematic review registration:**

https://www.crd.york.ac.uk/PROSPERO/, identifier: CRD42024607212.

## 1 Introduction

Diffuse large B-cell lymphoma (DLBCL) is the most common subtype of non-Hodgkin lymphoma (NHL), accounting for approximately 30%−40% of newly diagnosed NHL cases (1). Although many patients achieve favorable responses to first-line immunochemotherapy, treatment outcomes remain heterogeneous and are influenced by multiple clinical and biological factors (2). In routine practice, the doses of most immunochemotherapeutic agents are calculated according to body surface area, which is derived from actual body weight (3). However, excess body weight may affect body surface area–based dose estimation and alter the distribution and metabolism of lipophilic drugs, thereby influencing treatment efficacy and toxicity (4, 5). Accordingly, BMI and obesity-related body weight status have attracted increasing attention as potential prognostic factors in DLBCL.

Obesity has become an increasingly important challenge in oncology care because of its rising global prevalence and its potential impact on cancer risk, treatment response, and clinical outcomes, including in DLBCL (6, 7). Among the available measures of obesity, body mass index (BMI) remains the most widely used in clinical practice (8). Accumulating evidence suggests that BMI may be associated with clinical outcomes in DLBCL, although findings remain inconsistent. Some studies have reported better survival among overweight or obese patients (9, 10), whereas others have suggested that this survival benefit may be limited to the overweight group, with no statistically significant association observed for obesity (11). For example, one retrospective cohort study reported better overall survival (OS) and progression-free survival (PFS) in overweight patients, whereas underweight and morbidly obese patients had poorer outcomes (12). In contrast, a cooperative study of elderly patients aged 60 years or older did not identify a significant association between BMI and survival (13).

More than 640 million adults worldwide were already classified as obese about a decade ago, and projections suggest that this number may rise to 1.02 billion by 2030, accounting for 18% of the global adult population (14, 15). Given the increasing global burden of obesity and the inconsistent findings reported in DLBCL, we conducted a systematic review and meta-analysis to evaluate the prognostic significance of BMI in patients with DLBCL, with a particular focus on OS and PFS.

## 2 Methods

This systematic review and meta-analysis was conducted in accordance with the PRISMA statement and was registered with PROSPERO (ID: CRD42024607212)(16).

### 2.1 Data sources and search strategy

To identify eligible studies, the Cochrane Library, PubMed, and Embase were systematically searched from database inception to April 14, 2026. Only English-language publications were considered. The search strategy combined database-specific controlled vocabulary terms, where available, with free-text terms to improve search sensitivity and coverage. In addition, backward reference screening of the included studies was performed to identify further relevant articles (17). Detailed search strategies are provided in Supplementary Appendix 1.

### 2.2 Study selection

Three independent reviewers (LS, Y-j T, and Y-c Z) screened titles and abstracts for potential eligibility and then assessed the full texts according to predefined criteria. Disagreements during study selection or data extraction were resolved through discussion with a fourth reviewer (JW). All reviewers underwent training before screening to standardize procedures and ensure consistent application of the eligibility criteria. Studies were included if they: (1) enrolled patients with pathologically confirmed DLBCL diagnosed according to internationally accepted criteria; (2) examined the association between BMI and survival outcomes; and (3) reported OS and/or PFS. Studies were excluded if they: (1) did not provide sufficient data to estimate or interpret hazard ratios (HRs); or (2) involved overlapping patient populations, in which case only the most recent or most comprehensive report was retained.

### 2.3 Data extraction and quality assessment

Information extracted from each eligible study included the author, country, sample size, participant characteristics including age and sex, treatment regimen, study design, duration of follow-up, BMI classification method, and survival endpoints. Methodological quality was assessed using the Methodological Index for Non-Randomized Studies (MINORS) tool (18). Each item was scored from 0 to 2, with a maximum total score of 16. Two reviewers (Y-j T and Y-c Z) performed the assessment independently, and any discrepancies were resolved through discussion with a third reviewer (JW).

### 2.4 Exposure definition

Conventional WHO BMI categories were defined as underweight (< 18.5 kg/m^2^), normal weight (18.5– < 25.0 kg/m^2^), overweight (25.0– < 30.0 kg/m^2^), and obesity (≥30.0 kg/m^2^) (19). For Asian populations, lower BMI thresholds have been recommended (20, 21). Accordingly, Asian-specific BMI frameworks generally apply lower thresholds, with 23.0 kg/m^2^ commonly used as the cutoff for overweight or elevated BMI in East Asian populations (19). BMI definitions were extracted according to the criteria used in the original studies. Because individual-level data were unavailable, we retained the BMI categories as defined in each original study and did not reclassify participants according to a uniform BMI framework.

### 2.5 Statistical analysis

To evaluate the association between BMI and survival outcomes in DLBCL, pooled HRs and corresponding 95% CIs were calculated (22). When HRs were not directly reported, Kaplan-Meier curves were digitized and pseudo-individual patient data were reconstructed using the IPDfromKM R package (23). Given the anticipated clinical and methodological heterogeneity across studies, all primary and subgroup meta-analyses were performed using the DerSimonian-Laird random-effects model (24), which accounts for between-study variability and generally provides more conservative estimates than a fixed-effects model. Between-study heterogeneity was assessed using Cochran's *Q*-test and the *I*^2^ statistic, with *I*^2^ values ≥50% considered to indicate substantial heterogeneity. Sensitivity analyses were conducted by sequentially omitting individual studies (18). Because the R-CHOP regimen, consisting of rituximab, cyclophosphamide, doxorubicin, vincristine, and prednisone, remains the cornerstone of first-line therapy for DLBCL, a subgroup analysis restricted to R-CHOP-like regimens was performed (1). Additional subgroup analyses were conducted according to BMI category, multivariable adjustment status, and use of normal-weight patients as the reference group. When at least 10 studies were available, potential reporting bias was assessed using funnel plots and Egger's test (25). All statistical analyses were performed using Stata, and a two-sided *P*-value < 0.05 was considered statistically significant.

## 3 Results

### 3.1 Study screening and inclusion

**Figure 1:**
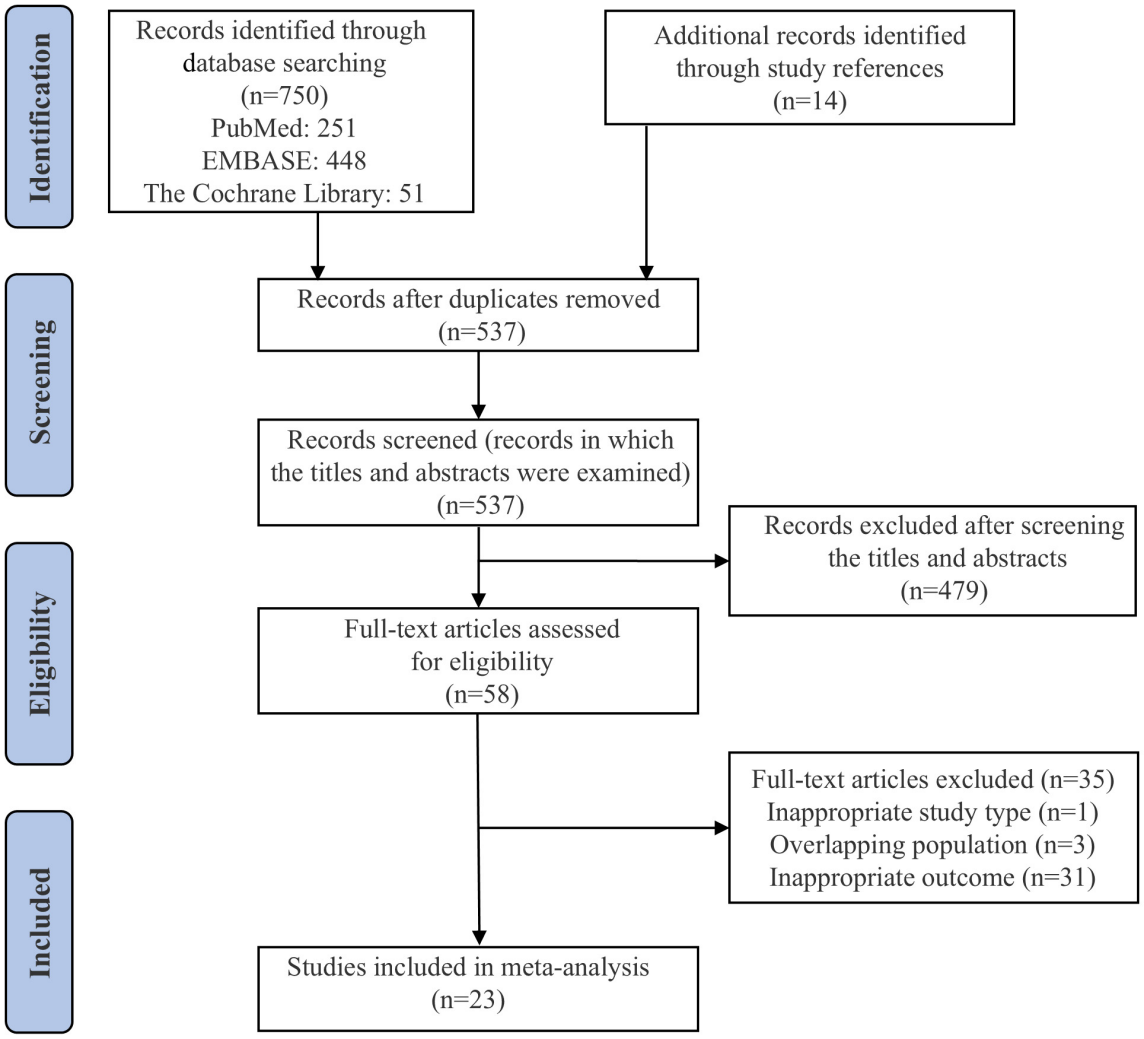
Flow diagram for the study selection process.

The study selection process is shown in Figure 1. A total of 764 records were identified, including 750 through database searching and 14 through reference screening. After duplicate removal, 537 records underwent title and abstract screening, of which 479 were excluded. 58 full-text articles were assessed for eligibility. Ultimately, 23 studies were included in the meta-analysis (9–13, 26–43). Excluded studies and the reasons for exclusion are listed in Supplementary Appendix 2.

### 3.2 Study characteristics and quality assessment

The main characteristics of the included studies are summarized in Table 1. Overall, 23 studies were included. The studies were published between 2010 and 2026 and were conducted in the United States (*n* = 6) (9, 11, 13, 26–28), France (*n* = 5) (29–33), Korea (*n* = 3) (12, 34, 35), China (*n* = 2) (36, 37), and Austria (10), Canada (38), Denmark (39), Italy (40), Portugal (41), Sweden (42), and Australasia (43). Most studies were retrospective (*n* = 18), and five were prospective (13, 26, 31, 32, 43). Where reported, follow-up duration ranged from 25 to 180 months. Treatment was mainly based on rituximab-containing or R-CHOP-like regimens, and one study involved CAR-T therapy (29). Sungur et al. reported survival data only for underweight patients, without providing specific data for overweight or obese patients (43). In addition, the BMI-stratified survival curves overlapped and had limited resolution, restricting data extraction using the IPDfromKM package. Consequently, only the underweight subgroup was included in this meta-analysis. Only one included study reconstructed survival data from digitized Kaplan-Meier curves using the IPDfromKM package (40).

**Table 1:** Baseline characteristics of the included studies.

Study	Year	Location	Sample size	Age	Sex (M/F)	Treatment	Study design	Follow-up	Outcome	Quality
Bendtsen et al. (39)	2017	Denmark	653	66.3 (57.5–74.2)	369/284	R-CHOP-like regimens	retrospective	69.6	PFS, OS	14
Besutti et al. (40)	2021	Italy	116	63.7 ± 16.4	60/56	R-CHOP-like regimens	retrospective	30 (24–34)	PFS, OS	13
Boyle et al. (38)	2017	Canada	238	59	154/84	Rituximab	retrospective	180	PFS, OS	12
Carson et al. (9)	2012	USA	2272	68 (20–100)	2469/65	Rituximab	retrospective	60	OS	10
Chihara et al. (26)	2021	USA	670	63 (18–92)	369/301	NR	prospective	108 (83–143)	PFS, OS	11
Coutinho et al. (41)	2019	Portugal	321	64 (16–89)	189/197	R-CHOP	retrospective	59	PFS, OS	11
Detroit et al. (29)	2023	France	132	63 (55–69.2)	82/50	CAR T^*^	retrospective	NR	PFS	12
Geyer et al. (27)	2010	USA	420	58 (20–74)	231/189	NR	retrospective	92.4	OS	10
Go et al. (34)	2019	Korea	228	64 (21–88)	130/98	R-CHOP	retrospective	76	PFS, OS	11
Hong et al. (13)	2014	USA	537	70 (60–92)	270/267	R-CHOP	prospective	112.8	OS	13
Jones et al. (11)	2010	USA	712	55.8	392/320	Chemotherapy regimens	retrospective	62.8	PFS, OS	11
Lanic et al. (30)	2014	France	82	78 (70–95)	36/46	R-CHOP-like regimens	retrospective	39	PFS, OS	14
Li et al. (36)	2016	China	143	60	73/70	R-CHOP-like regimens	retrospective	NR	OS	10
Mörth et al. (42)	2019	Sweden	612	66.1 (18–91)	358/254	R-CHOP, CHOP	retrospective	NR	OS	12
Pénichoux et al. (31)	2023	France	95	78.4 (70–94)	47/48	R-CHOP	prospective	NR	PFS, OS	12
Sarkozy et al. (32)	2014	France	985	68	533/452	R-CHOP	prospective	NR	PFS, OS	10
Shin et al. (35)	2016	Korea	156	61 (27–68)	81/75	R-CHOP	retrospective	NR	PFS, OS	9
Weiss et al. (10)	2014	Austria	183	66.5 ± 15.3	102/81	R-CHOP-like regimens	retrospective	44	OS	11
Yang et al. (37)	2021	China	131	NR	65/66	R-CHOP-like regimens	retrospective	60.36	PFS, OS	11
Zhou et al. (28)	2016	USA	1386	58	749/637	Rituximab, chemotherapy	retrospective	NR	PFS, OS	12
Penichoux et al. (33)	2025	France	219	32 (18–88)	90/129	R-CHOP-like regimens	retrospective	51	PFS, OS	10
Hwang et al. (12)	2015	Korea	359	54 (16–85)	313/249	R-CHOP-like regimens	retrospective	46	PFS, OS	10
Sungur et al. (43)	2026	Australasia	933	NR	NR	R-CHOP and others	prospective	25	PFS, OS	11

Age (years) and follow-up (months) were reported as median (range). R-CHOP, rituximab, cyclophosphamide, doxorubicin, vincristine and prednisolone. NR, not reported. OS, overall survival. PFS, progression-free survival.

Seventeen studies reported both OS and PFS, five reported OS only (13, 26, 31, 32, 43), and one reported PFS only (29). Overall, 22 studies involving 11,451 patients reported OS-related data, and 17 studies involving 7,416 patients reported PFS data. Sungur et al. provided extractable estimates only for underweight patients (43). Accordingly, the primary OS analysis and the corresponding assessment of publication bias included 21 studies involving 10,518 patients. For PFS, after excluding Sungur et al. for the same reason and one additional study evaluating CAR T-cell therapy, the primary PFS analysis and publication bias assessment included 15 studies involving 6,351 patients (29, 43). Among the included studies, five studies conducted in Asian populations used an Asian-specific BMI cutoff of 23 kg/m^2^ to define overweight (12, 34–37), whereas the remaining studies applied the conventional WHO BMI criterion, with a cutoff of 25 kg/m^2^. Detailed information is presented in Supplementary Table 1. Study quality was assessed using the MINORS tool. Scores ranged from 9 to 14, with a median of 11, indicating overall moderate-to-good methodological quality. Individual scoring results are shown in Table 1.

### 3.3 Prognostic value of BMI for OS

**Figure 2:**
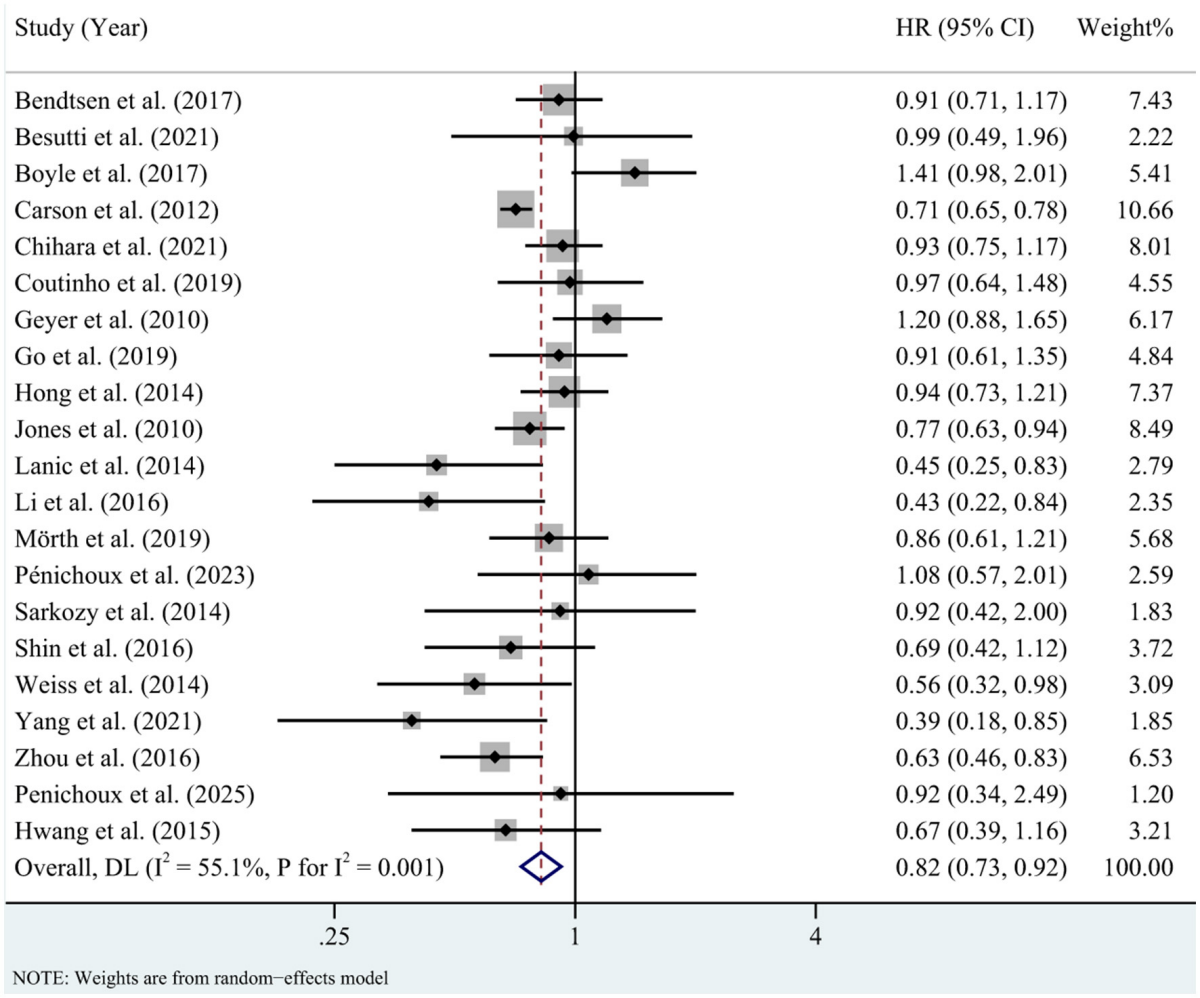
Forest plot of the meta-analysis evaluating the association between overweight/obesity and OS in patients with DLBCL, compared with patients without overweight/obesity (hazard ratios). CI, confidence interval; DL, DerSimonian-Laird estimate; *I*^2^, inconsistency; *P* for *I*^2^, *P*-value for the test of between-study heterogeneity.

Twenty-one studies including 10,518 patients compared the difference in OS between patients with overweight or obesity and patients without overweight or obesity. The pooled result showed that patients with overweight or obesity had better OS (HR = 0.82; 95% CI: 0.73–0.92, *P* = 0.001), with moderate heterogeneity across studies (*I*^2^ = 55.1%, *P* for heterogeneity = 0.001). The study that reconstructed survival data using the IPDfromKM package contributed only a small proportion of the overall weight (2.22%) in the OS analysis (40) (Figure 2). Leave-one-out sensitivity analysis showed that no single study materially influenced the pooled estimate (Supplementary Figure 1).

### 3.4 Subgroup analysis of OS

This association was consistent across subgroup analyses. In studies restricted to R-CHOP-like regimens, overweight or obesity remained associated with better OS (15 studies involving 4,820 patients; HR = 0.80; 95% CI: 0.70–0.92, *P* = 0.001), with low heterogeneity (*I*^2^ = 20.5%, *P* for heterogeneity = 0.225) (Supplementary Figure 2). This association was also observed when the analysis was limited to multivariable-adjusted estimates (16 studies involving 9,778 patients; HR = 0.82; 95% CI: 0.72–0.93, *P* = 0.002), although heterogeneity remained moderate (*I*^2^ = 61.7%, *P* for heterogeneity < 0.001) (Supplementary Figure 3). When normal-weight patients were used as the reference group, the pooled effect estimate remained similar (11 studies involving 8,369 patients; HR = 0.82; 95% CI: 0.73–0.93, *P* = 0.001), with moderate heterogeneity (*I*^2^ = 50.2%, *P* for heterogeneity = 0.029) (Supplementary Figure 4).

**Figure 3:**
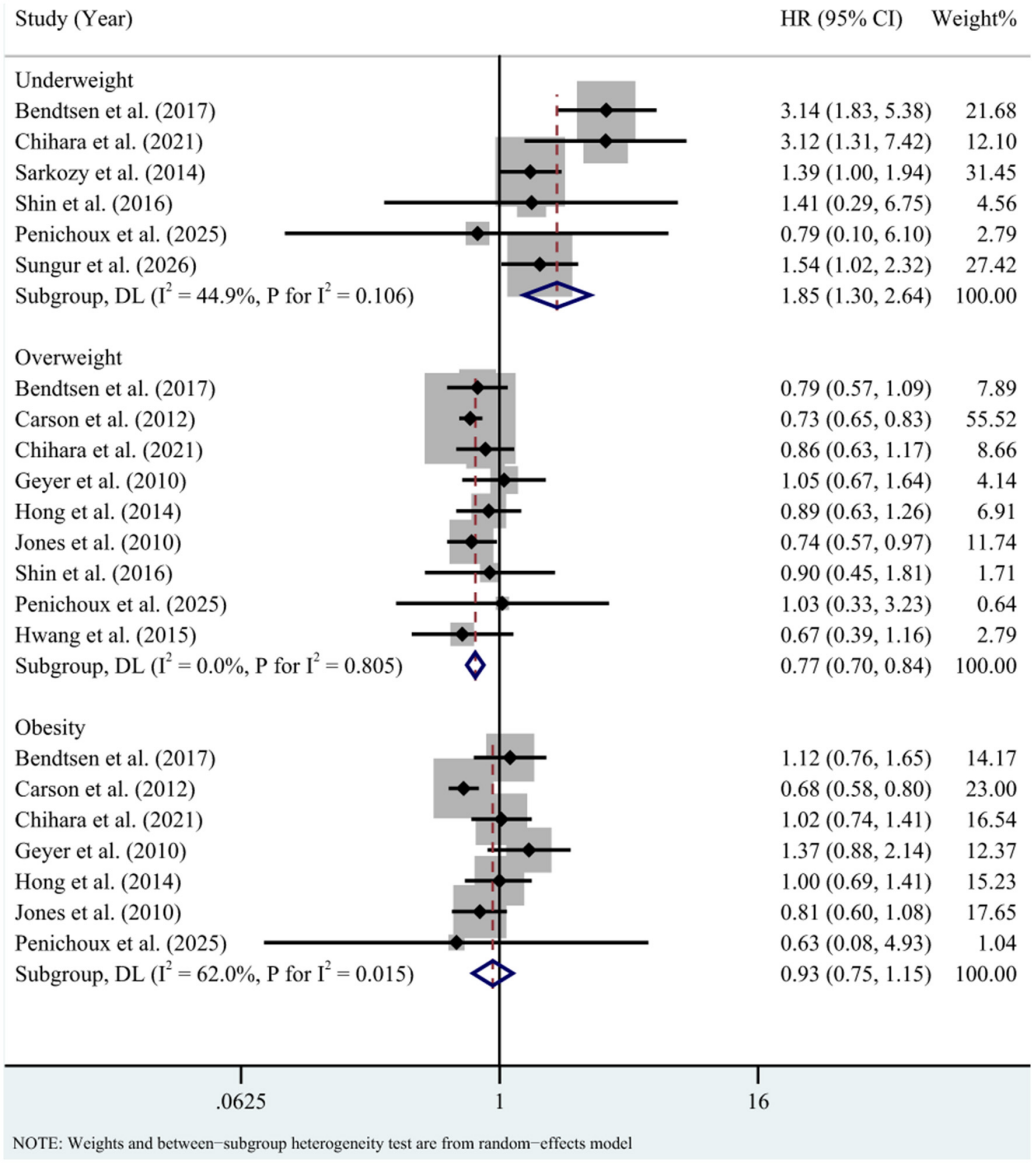
Forest plot of the BMI category-specific subgroup analysis evaluating the association between BMI category and OS in patients with DLBCL (hazard ratios). CI, confidence interval; DL, DerSimonian-Laird estimate; I^2^, inconsistency; *P* for *I*^2^, *P*-value for the test of between-study heterogeneity.

Further analysis by BMI category showed distinct associations across BMI categories (Figure 3). Underweight status was associated with inferior OS (6 studies involving 3,616 patients; HR = 1.85; 95% CI: 1.30–2.64, *P* = 0.001), with moderate heterogeneity (*I*^2^ = 44.9%, *P* for heterogeneity = 0.106). In contrast, overweight status was associated with better OS (9 studies involving 5,989 patients; HR = 0.77; 95% CI: 0.70–0.84, *P* < 0.001), with no evidence of heterogeneity (*I*^2^ = 0.0%, *P* for heterogeneity = 0.805). Obesity was not significantly associated with OS (7 studies involving 5,474 patients; HR = 0.93; 95% CI: 0.75–1.15, *P* = 0.508), and heterogeneity was substantial (*I*^2^ = 62.0%, *P* for heterogeneity = 0.015).

### 3.5 Prognostic value of BMI for PFS

A total of 16 studies involving 6,483 patients compared the difference in PFS between patients with overweight or obesity and patients without overweight or obesity. The pooled result showed that patients with overweight or obesity had better PFS (HR = 0.83; 95% CI: 0.72–0.95, *P* = 0.009), with moderate between-study heterogeneity (*I*^2^ = 53.5%, P for heterogeneity = 0.006) (Supplementary Figure 5). Because one study involved CAR-T therapy rather than conventional first-line immunochemotherapy (29), it was excluded from the primary pooled analysis owing to the marked difference in treatment setting.

**Figure 4:**
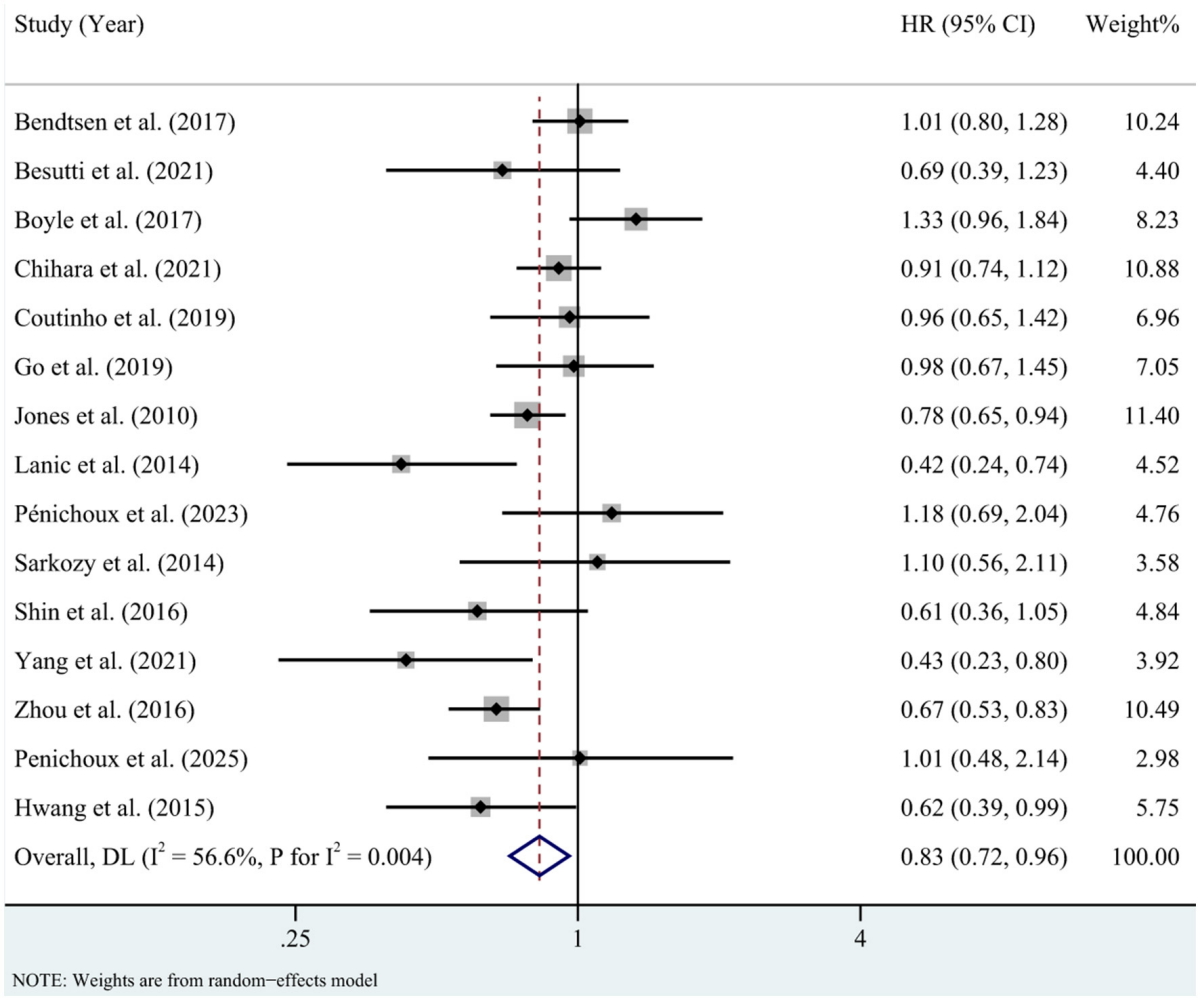
Forest plot of the meta-analysis evaluating the association between overweight/obesity and PFS in patients with DLBCL, compared with patients without overweight/obesity (hazard ratios). CI, confidence interval; DL, DerSimonian-Laird estimate; *I*^2^, inconsistency; *P* for *I*^2^, *P*-value for the test of between-study heterogeneity.

The primary PFS analysis therefore included 15 studies involving 6,351 patients. Overall, compared with patients without overweight or obesity, patients with overweight or obesity showed better PFS (HR = 0.83; 95% CI: 0.72–0.96, *P* = 0.011), with moderate between-study heterogeneity (*I*^2^ = 56.6%, *P* for heterogeneity = 0.004) (Figure 4). The study that reconstructed survival data using the IPDfromKM package contributed only a small proportion of the overall weight (4.40%) in the PFS analysis (40). Leave-one-out sensitivity analysis showed that no single study materially influenced the pooled estimate, and the overall association remained stable after sequential omission of each study (Supplementary Figure 6).

### 3.6 Subgroup analysis of PFS

This association was maintained across subgroup analyses. In studies restricted to R-CHOP-like regimens, overweight or obesity remained associated with better PFS (10 studies involving 3,126 patients; HR = 0.81; 95% CI: 0.66–0.99, *P* = 0.042), with moderate heterogeneity (*I*^2^ = 51.7%, *P* for heterogeneity = 0.023) (Supplementary Figure 7). A similar association was observed when only multivariable-adjusted estimates were included (10 studies involving 5,588 patients; HR = 0.83; 95% CI: 0.71–0.98, *P* = 0.029), although heterogeneity remained moderate (*I*^2^ = 61.9%, *P* for heterogeneity = 0.005) (Supplementary Figure 8). When normal-weight patients were used as the reference group, the pooled HR was 0.81 (eight studies involving 5,117 patients; 95% CI: 0.71–0.93, *P* = 0.003), with lower heterogeneity (*I*^2^ = 36.3%, *P* for heterogeneity = 0.139) (Supplementary Figure 9).

**Figure 5:**
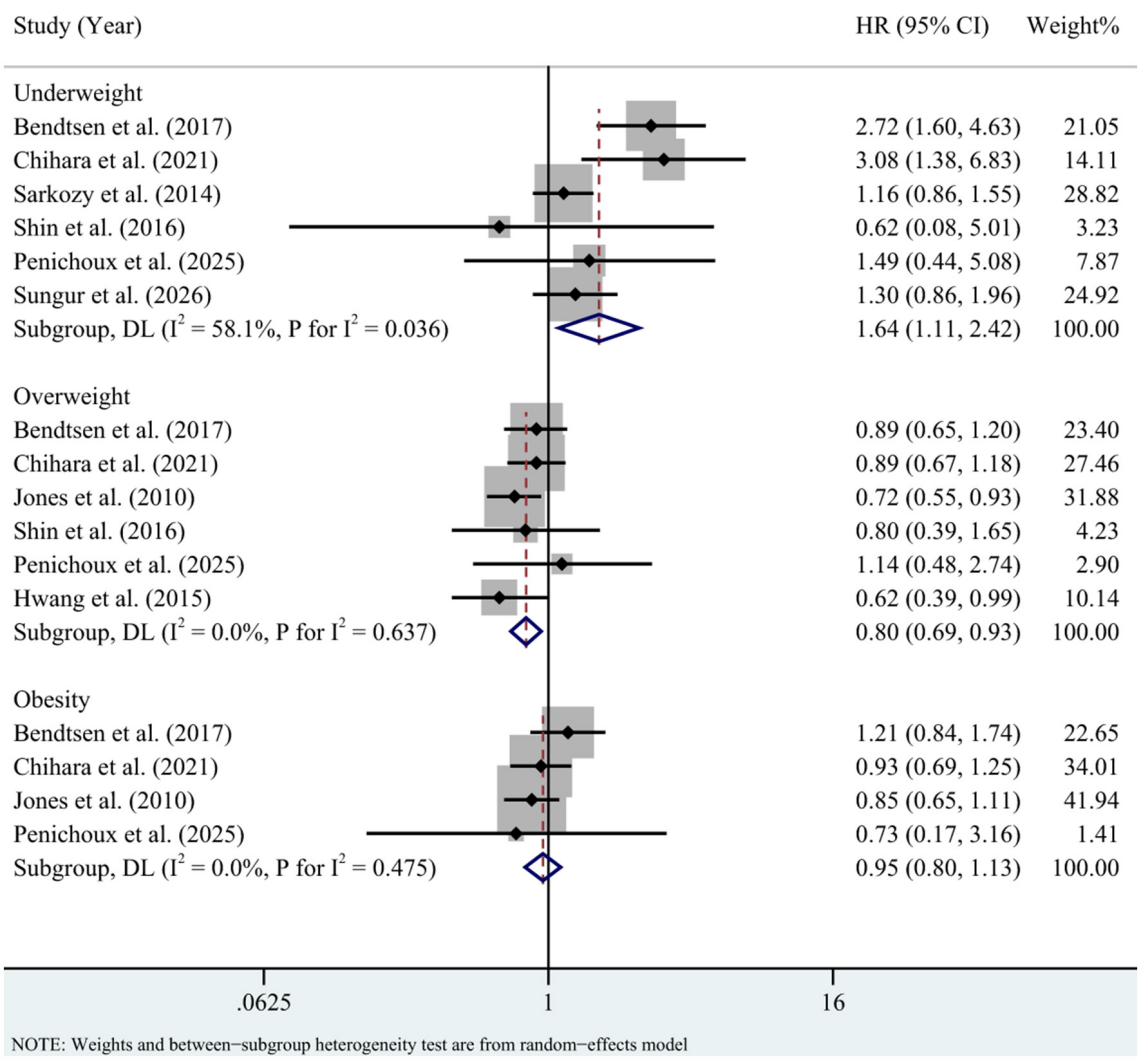
Forest plot of the BMI category-specific subgroup analysis evaluating the association between BMI category and PFS in patients with DLBCL (hazard ratios). CI, confidence interval; DL, DerSimonian-Laird estimate; *I*^2^, inconsistency; *P* for *I*^2^, *P*-value for the test of between-study heterogeneity.

Further analysis by BMI category showed distinct patterns across BMI groups (Figure 5). Underweight status was associated with inferior PFS (6 studies involving 3,616 patients; HR = 1.64; 95% CI: 1.11–2.42, *P* = 0.013), with moderate heterogeneity (*I*^2^ = 58.1%, *P* for heterogeneity = 0.036). In contrast, overweight status was associated with better PFS (6 studies involving 2,746 patients; HR = 0.80; 95% CI: 0.69–0.93, *P* = 0.004), with no evidence of heterogeneity (*I*^2^ = 0.0%, *P* for heterogeneity = 0.637). Obesity was not significantly associated with PFS (4 studies involving 2,231 patients; HR = 0.95; 95% CI: 0.80–1.13, *P* = 0.541), and no between-study heterogeneity was observed (*I*^2^ = 0.0%, *P* for heterogeneity = 0.475).

### 3.7 Publication bias

Potential reporting bias was assessed using funnel plots and Egger's tests. For OS, the funnel plot based on the 21 studies included in the OS meta-analysis showed no marked asymmetry (Supplementary Figure 10), and Egger's test showed no evidence of significant publication bias (*P* = 0.514; Supplementary Figure 11). For PFS, the funnel plot based on the 15 studies included in the primary PFS meta-analysis showed no marked asymmetry (Supplementary Figure 12), and Egger's test showed no evidence of significant publication bias (*P* = 0.628; Supplementary Figure 13).

## 4 Discussion

In the present meta-analysis, overweight or obesity was consistently associated with better survival outcomes in patients with DLBCL, including both OS and PFS. This pattern remained generally stable across clinically relevant subgroup analyses, including analyses restricted to R-CHOP-like regimens, analyses based on multivariable-adjusted estimates, and analyses using normal-weight patients as the reference group. Further analyses by BMI category showed that the apparent survival advantage was most evident among patients with overweight, whereas underweight status was associated with poorer OS and PFS. In contrast, obesity was not significantly associated with OS or PFS, suggesting that the observed association for overweight or obesity combined may have been primarily driven by the overweight subgroup. Current evidence remains insufficient to confirm a meaningful prognostic impact of obesity in DLBCL.

Compared with the previous meta-analysis by Wang et al. (44), the present study provides a more updated and clinically informative synthesis of the association between BMI and prognosis in DLBCL. First, compared with the previous meta-analysis, the current study substantially expanded the evidence base, with the number of included studies increasing from 14 to 23. In the present analysis, 22 studies involving 11,451 patients were available for OS, and 17 studies involving 7,416 patients were available for PFS. In addition, the literature search was updated to April 14, 2026. Wang et al. searched PubMed, Web of Science, EMBASE, and the Cochrane Library, whereas the current study searched PubMed, EMBASE, and the Cochrane Library and further supplemented the search through backward reference screening. The larger evidence base and updated analysis also yielded a more comprehensive outcome profile. In the meta-analysis by Wang et al., overweight was associated with better OS, whereas no significant association was observed for PFS. By contrast, in the current study, overweight was associated with both better OS and better PFS. Second, BMI definitions in East Asian populations were addressed more explicitly. WHO recommendations and subsequent regional consensus statements support the use of lower BMI thresholds in Asian populations than in Western settings (20, 21). For example, in Go et al., the same East Asian cohort was analyzed using both Asian (BMI ≥23 kg/m^2^) and Western (BMI ≥25 kg/m^2^) definitions; although the direction of association remained unchanged, the effect estimates differed, suggesting that cutoff selection may influence the observed effect size. In contrast to Wang et al., our updated analysis included East Asian studies that applied Asian-appropriate BMI definitions, which improves the methodological coherence and interpretability of the pooled estimates in Asian populations. Finally, we performed additional clinically relevant subgroup analyses according to treatment regimen (R-CHOP-like regimens), multivariable adjustment status, reference group definition (studies using normal-weight patients as the reference group), and BMI category, thereby better defining the settings in which BMI may have prognostic relevance.

Our research indicated that even after controlling for known confounding factors, overweight patients consistently demonstrated superior survival outcomes. During immunochemotherapy, the drug dosage is traditionally determined based on body surface area relative to actual body weight (3). The various biological and pharmacological changes induced by the overweight status may help explain these findings. One consideration is that the lipophilic nature of anthracyclines like doxorubicin enables a wider tissue distribution in patients with more adipose tissue, potentially increasing effective drug exposure (4, 45). Second, overweight-related hepatic metabolic alterations (such as steatosis and changes in hepatic perfusion) may reduce drug clearance rates, thereby extending systemic drug activity and enhancing therapeutic efficacy, which has been linked to better prognosis (45–47). Third, nutritional and physiological reserve may also play a role. Individuals with higher BMI typically have greater nutritional and energy reserves, which may improve treatment tolerance and long-term outcomes (48, 49). Additionally, another explanation involves differences between pro-tumor and anti-tumor immune cells. Notably, the immune system takes on a pivotal role in the prognosis and treatment response of DLBCL. It has been reported that overweight DLBCL patients exhibit elevated levels of NK-like T cells, whose cytotoxic activity may contribute to antitumor immune responses and enhance treatment efficacy (50).

Our research indicated that overweight status correlated with superior survival outcomes, whereas obesity lacked significant prognostic relevance. This suggests that the relationship between BMI and DLBCL prognosis may be nonlinear, potentially even exhibiting an inverted U-shaped curve. Actually, nonlinear associations between obesity and other tumor prognoses have been extensively reported (51). Several interconnected mechanisms may explain this pattern. First, the metabolic and compositional distinctions between overweight and obese individuals are substantial. While overweight status may preserve muscle mass, obesity typically involves excessive visceral and intermuscular fat accumulation accompanied by sarcopenia (35). This shift may lead to a pro-inflammatory state in DLBCL patients, reducing treatment efficacy (35, 52). Additionally, the interaction between tumors and adipose tissue may also influence the efficacy of chemotherapy. Experimental data indicate that adipocytes under oxidative stress can protect malignant cells from anthracycline-induced damage (53, 54). Beyond local effects, systemic complications may also mediate worse outcomes. Obesity exacerbates cardiotoxic risks associated with anthracycline exposure, as evidenced by cardiac dysfunction in pediatric and adult lymphoma survivors with excess body fat at diagnosis (5, 55, 56). At the molecular level, expanded white adipose tissue operates as an active endocrine organ, secreting adipokines, lipids, and metabolites that influence the tumor milieu (57–61). For example, adiponectin has been found to dampen immune activation by inhibiting apoptotic signaling (62, 63). Additionally, 20 years ago, Gruberg et al. reported the “obesity paradox” in cardiovascular disease, namely that individuals with elevated BMI demonstrated unexpectedly better survival (64). Notably, the obese patients in Gruberg et al.‘s study were significantly younger. Given the current widespread recognition that obesity promotes tumor development, obese patients with DLBCL may also be younger, thereby benefiting from tumor treatment (51). These may partially explain our results, analogous to the process of removing weights to achieve an approximate equilibrium on a tilted balance.

Regarding outcomes in obesity patients, another factor to consider is the actual intensity of treatment. Relative dose intensity (RDI) refers to the amount of drug delivered per unit time relative to the amount planned in the standard treatment schedule (65–67). Previous studies have suggested that maintaining a higher RDI during first-line treatment is associated with better outcomes in DLBCL( 66–68). Doses in most first-line regimens are calculated according to body surface area derived from actual body weight (3). However, although higher body weight may increase the absolute administered dose, the actual delivered dose intensity may still vary substantially in clinical practice. In patients with obesity, concerns about toxicity and other clinical factors may lead to dose reduction, dose capping, treatment delay, or early discontinuation, thereby resulting in lower RDI (51, 68–70). Accordingly, the association between BMI and prognosis may reflect not only biological and pharmacokinetic factors but also differences in delivered dose intensity. Although direct evidence linking BMI and RDI in DLBCL and other hematologic malignancies remains limited, studies in breast cancer suggest that BMI may influence chemotherapy dose-modification patterns (70), highlighting the need to consider this issue in lymphoma as well. Because relevant data on delivered dose intensity were unavailable, this potential source of confounding could not be evaluated in the present meta-analysis. Future studies examining the association between BMI and prognosis in DLBCL should therefore collect and adjust for actual delivered dose intensity. Together, these factors may help explain why the apparent survival advantage observed in overweight patients was not maintained in the obesity group.

Individuals at the lower end of the BMI spectrum exhibited significantly worse OS, although PFS did not show a clear statistical relationship. Several interconnected factors may account for this observation. One hypothesis is that limited adipose reserves in underweight patients restrict the distribution of lipophilic agents such as anthracyclines, thereby reducing systemic exposure and diminishing treatment efficacy (4, 45, 71, 72). Beyond pharmacokinetics, body composition plays a crucial role. Underweight status often coincides with reductions in both fat stores and lean muscle mass, characteristics of tumor-induced cachexia (73, 74). This metabolic syndrome is linked to lower functional capacity, reduced treatment tolerance, and attenuated immune response—all contributing to inferior clinical outcomes (75, 76). Treatment complications also appear more frequently in these patients. A prospective study revealed impaired cardiac function in individuals with low body weight, independent of prior therapy (77). Compared to heart failure patients, cancer survivors showed more pronounced loss of ventricular mass, diminished physical performance, and increased mortality risk, especially among those with cachexia (77). These outcomes likely stem from severe malnutrition, leading to myocardial fibrosis, protein catabolism, and structural deterioration of cardiac tissue (78, 79).

To explore the potential sources of heterogeneity among the pooled analyses, we conducted predefined subgroup analyses according to treatment regimen, multivariable adjustment status, use of normal-weight patients as the reference group, and BMI category. Reduced heterogeneity was observed in several subgroup analyses. For example, in the OS analysis, heterogeneity reduced significantly when the analysis was restricted to R-CHOP-like regimens. Similarly, in the PFS analysis, heterogeneity also decreased significantly when the reference group was limited to normal-weight patients. In addition, no heterogeneity was observed in BMI category-specific analyses for the overweight category in OS and for both the overweight and obesity categories in PFS, suggesting that differences in BMI categorization may also have contributed to the heterogeneity observed in the primary analyses. Nevertheless, residual heterogeneity persisted in several other subgroup analyses, which may be attributed to variations among studies in treatment duration, follow-up duration, baseline patient characteristics, and study design.

Although this meta-analysis provides meaningful insights, several limitations should be acknowledged. First, because of the structure and reporting of the available data, BMI could only be analyzed as a categorical rather than a continuous variable. This limited our ability to explore potential nonlinear associations with DLBCL prognosis. In addition, the original data did not allow us to assess the prognostic impact of BMI changes during treatment. Second, the included studies used different BMI classification systems, including conventional WHO-based thresholds and Asian-specific cutoffs. Because individual-level data were unavailable, we were unable to reclassify participants using a unified BMI framework, which implied that the possibility of systematic misclassification bias cannot be ruled out. Third, several BMI category-specific subgroup analyses, particularly those for underweight and obesity, were based on a limited number of studies. This may have reduced statistical power and contributed to the wide confidence intervals observed for some estimates. These findings should therefore be interpreted with caution. Fourth, effect estimates from one included study, Besutti et al. (40), were derived through reconstruction of digitized Kaplan-Meier curves using the IPDfromKM package. This approach may introduce measurement error related to image quality and the curve digitization process. However, this study contributed only a small proportion of the overall evidence. Therefore, the potential measurement error introduced by data reconstruction is unlikely to have materially influenced the overall pooled estimates. Fifth, because relevant data on RDI were unavailable, we were unable to evaluate its potential confounding effect. Finally, much of the available evidence was derived from retrospective studies, and our analyses therefore primarily relied on this study design. Future prospective studies with detailed clinical data are needed to evaluate BMI as a continuous variable, apply time-dependent models during treatment, and adjust for actual delivered dose intensity when assessing the association between BMI and prognosis, thereby better defining the prognostic significance of BMI in DLBCL.

## 5 Conclusions

Given its simplicity and routine availability, BMI may serve as a practical adjunctive indicator in the baseline assessment of patients with DLBCL. Our findings suggest that patients with overweight or obesity may have better survival outcomes than those without overweight or obesity, with the most evident benefit observed in the overweight category, whereas underweight status appears to be associated with poorer prognosis. Current evidence remains insufficient to confirm a significant prognostic impact of obesity. In light of the limitations of the present study, further prospective studies are warranted to evaluate BMI as a continuous variable and to incorporate time-dependent modeling during treatment to better define its relationship with DLBCL outcomes.

## Data Availability

The original contributions presented in the study are included in the article/supplementary material, further inquiries can be directed to the corresponding authors.

## References

[1] Susanibar-Adaniya S Barta SK. 2021 Update on diffuse large B cell lymphoma: a review of current data and potential applications on risk stratification and management. Am J Hematol. (2021) 96:617–29. doi: 10.1002/ajh.26151 33661537 PMC8172085

[2] Bock AM Epperla N. Therapeutic landscape of primary refractory and relapsed diffuse large B-cell lymphoma: recent advances and emerging therapies. J Hematol Oncol. (2025) 18:68. doi: 10.1186/s13045-025-01702-5 40597378 PMC12217534

[3] Liang P Zhu MY Yang R Wang X Yue H Zheng Y . Obese patients with malignant tumor: a case series and literature review. Discov Oncol. (2025) 16:1020. doi: 10.1007/s12672-025-02689-8 40481221 PMC12143997

[4] Bruno CD Harmatz JS Duan SX Zhang Q Chow CR Greenblatt DJ. Effect of lipophilicity on drug distribution and elimination: influence of obesity. Br J Clin Pharmacol. (2021) 87:3197–205. doi: 10.1111/bcp.14735 33450083

[5] George IA Souder B Berkman A Noyd DH Jay Campbell M Barker PCA . Obesity predisposes anthracycline-treated survivors of childhood and adolescent cancers to subclinical cardiac dysfunction. Pediatr Cardiol. (2024) 46:362–71. doi: 10.1007/s00246-024-03423-x 38456890 PMC11380701

[6] Wen X Zhang B Wu B Xiao H Li Z Li R . Signaling pathways in obesity: mechanisms and therapeutic interventions. Signal Transduct Target Ther. (2022) 7:298. doi: 10.1038/s41392-022-01149-x 36031641 PMC9420733

[7] Jin X Qiu T Li L Yu R Chen X Li C . Pathophysiology of obesity and its associated diseases. Acta Pharm Sin B. (2023) 13:2403–24. doi: 10.1016/j.apsb.2023.01.012 37425065 PMC10326265

[8] Celind J Ohlsson C Bygdell M Martikainen J Lewerin C Kindblom JM. Childhood body mass index is associated with the risk of adult hematologic malignancies in men-the best Gothenburg cohort. Int J Cancer. (2020) 147:2355–62. doi: 10.1002/ijc.33015 32306396

[9] Carson KR Bartlett NL McDonald JR Luo S Zeringue A Liu J . Increased body mass index is associated with improved survival in United States veterans with diffuse large B-cell lymphoma. J Clin Oncol. (2012) 30:3217–22. doi: 10.1200/JCO.2011.39.2100 22649138 PMC3434980

[10] Weiss L Melchardt T Habringer S Boekstegers A Hufnagl C Neureiter D . Increased body mass index is associated with improved overall survival in diffuse large B-cell lymphoma. Ann Oncol. (2014) 25:171–6. doi: 10.1093/annonc/mdt481 24299961

[11] Jones JA Fayad LE Elting LS Rodriguez MA. Body mass index and outcomes in patients receiving chemotherapy for intermediate-grade B-cell non-Hodgkin lymphoma. Leuk Lymphoma. (2010) 51:1649–57. doi: 10.3109/10428194.2010.494315 20807093

[12] Hwang HS Yoon DH Suh C Huh J. Body mass index as a prognostic factor in Asian patients treated with chemoimmunotherapy for diffuse large B cell lymphoma, not otherwise specified. Ann Hematol. (2015) 94:1655–65. doi: 10.1007/s00277-015-2438-4 26174908

[13] Hong F Habermann TM Gordon LI Hochster H Gascoyne RD Morrison VA . The role of body mass index in survival outcome for lymphoma patients: US intergroup experience. Ann Oncol. (2014) 25:669–74. doi: 10.1093/annonc/mdt594 24567515 PMC4433526

[14] Lingvay I Cohen RV Roux CWL Sumithran P. Obesity in adults. Lancet. (2024) 404:972–87. doi: 10.1016/S0140-6736(24)01210-8 39159652

[15] Ling J Chen S Zahry NR Kao TA. Economic burden of childhood overweight and obesity: a systematic review and meta-analysis. Obes Rev. (2023) 24:e13535. doi: 10.1111/obr.13535 36437105 PMC10078467

[16] Moher D Liberati A Tetzlaff J Altman DG. Preferred reporting items for systematic reviews and meta-analyses: the PRISMA statement. Bmj. (2009) 339:b2535. doi: 10.1136/bmj.b2535 19622551 PMC2714657

[17] Binda NC Lavareze L de Souza Vieira G da Costa Tincani P Tincani AJ Chone CT . Impact of elective cervical dissection on the prognosis of patients with oral squamous cell carcinoma cT1/T2N0: a systematic review and meta-analysis. Crit Rev Oncol Hematol. (2025) 217:104982. doi: 10.1016/j.critrevonc.2025.104982 41115617

[18] Peng B Lu J Guo H Liu J Li A. Regional citrate anticoagulation for replacement therapy in patients with liver failure: a systematic review and meta-analysis. Front Nutr. (2023) 10:1031796. doi: 10.3389/fnut.2023.1031796 36875829 PMC9977825

[19] Gilden AH Catenacci VA Taormina JM. Obesity. Ann Intern Med. (2024) 177:Itc65-itc80. doi: 10.7326/AITC202405210 38739920

[20] Consultation WE. Appropriate body-mass index for Asian populations and its implications for policy and intervention strategies. Lancet. (2004) 363:157–63. doi: 10.1016/S0140-6736(03)15268-3 14726171

[21] Tham KW Abdul Ghani R Cua SC Deerochanawong C Fojas M Hocking S . Obesity in South and Southeast Asia-a new consensus on care and management. Obes Rev. (2023) 24:e13520. doi: 10.1111/obr.13520 36453081 PMC10078503

[22] Jayedi A Soltani S Motlagh SZ Emadi A Shahinfar H Moosavi H . Anthropometric and adiposity indicators and risk of type 2 diabetes: systematic review and dose-response meta-analysis of cohort studies. Bmj. (2022) 376:e067516. doi: 10.1136/bmj-2021-067516 35042741 PMC8764578

[23] Liu N Zhou Y Lee JJ. IPDfromKM: reconstruct individual patient data from published Kaplan-Meier survival curves. BMC Med Res Methodol. (2021) 21:111. doi: 10.1186/s12874-021-01308-8 34074267 PMC8168323

[24] Zamani K Rostami P Darehbagh RR Afraie M Moradi Y. Hepatitis B and C virus infection and risk of multiple myeloma: a systematic review and meta-analysis. BMC Cancer. (2025) 25:998. doi: 10.1186/s12885-025-14420-5 40468263 PMC12135261

[25] Li W Xie Y Jiang L. Coffee and tea consumption on the risk of osteoporosis: a meta-analysis. Front Nutr. (2025) 12:1559835. doi: 10.3389/fnut.2025.1559835 40104819 PMC11913691

[26] Chihara D Larson MC Robinson DP Thompson CA Maurer MJ Casulo C . Body mass index and survival of patients with lymphoma. Leuk Lymphoma. (2021) 62:2671–8. doi: 10.1080/10428194.2021.1929956 34121594 PMC8771423

[27] Geyer SM Morton LM Habermann TM Allmer C Davis S Cozen W . Smoking, alcohol use, obesity, and overall survival from non-Hodgkin lymphoma: a population-based study. Cancer. (2010) 116:2993–3000. doi: 10.1002/cncr.25114 20564404 PMC2889918

[28] Zhou Z Rademaker AW Gordon LI LaCasce AS Crosby-Thompson A Vanderplas A . High body mass index in elderly patients with DLBCL treated with rituximab-containing therapy compensates for negative impact of male sex. J Natl Compr Canc Netw. (2016) 14:1274–81. doi: 10.6004/jnccn.2016.0136 27697981 PMC5531178

[29] Detroit M Collier M Beeker N Willems L Decroocq J Deau-Fischer B . Predictive factors of response to immunotherapy in lymphomas: a multicentre clinical data warehouse study (PRONOSTIM). Cancers. (2023) 15:4028. doi: 10.3390/cancers15164028 37627056 PMC10452259

[30] Lanic H Kraut-Tauzia J Modzelewski R Clatot F Mareschal S Picquenot JM . Sarcopenia is an independent prognostic factor in elderly patients with diffuse large B-cell lymphoma treated with immunochemotherapy. Leuk Lymphoma. (2014) 55:817–23. doi: 10.3109/10428194.2013.816421 23781925

[31] Pénichoux J Lanic H Thill C Ménard AL Camus V Stamatoullas A . Prognostic relevance of sarcopenia, geriatric, and nutritional assessments in older patients with diffuse large B-cell lymphoma: results of a multicentric prospective cohort study. Ann Hematol. (2023) 102:1811–23. doi: 10.1007/s00277-023-05200-x 37058153 PMC10260702

[32] Sarkozy C Mounier N Delmer A Van Hoof A Karsenti JM Fleck E . Impact of BMI and gender on outcomes in DLBCL patients treated with R-CHOP: a pooled study from the LYSA. Lymphoma. (2014) 2014:205215. doi: 10.1155/2014/205215

[33] Penichoux J Decazes P Rossi C Sesques P Haioun C Durot E . Impact of body composition on treatment toxicity and outcomes in patients with primary mediastinal large B-cell lymphoma. Hematol Oncol. (2025) 43:e70117. 40646709 10.1002/hon.70117 PMC12254528

[34] Go SI Park S Kang MH Kim HG Kim HR Lee GW. Clinical impact of prognostic nutritional index in diffuse large B cell lymphoma. Ann Hematol. (2019) 98:401–11. doi: 10.1007/s00277-018-3540-1 30413902

[35] Shin DY Kim A Byun BH Moon H Kim S Ko YJ . Visceral adipose tissue is prognostic for survival of diffuse large B cell lymphoma treated with frontline R-CHOP. Ann Hematol. (2016) 95:409–16. doi: 10.1007/s00277-015-2571-0 26658607

[36] Li T Liu ZG Liang PQ Wang HT. Can body mass index predict the outcome of diffuse large B-cell lymphoma? a single-center retrospective study in China. Leuk Lymphoma. (2017) 58:1624–9. doi: 10.1080/10428194.2016.1257793 27868453

[37] Yang Y Liu Z Zhang G Wang H. A concise prognostic score system for diffuse large B-cell lymphoma: a retrospective study with long-term follow-up. Future Oncol. (2021) 17:4299–306. doi: 10.2217/fon-2020-1073 34350771

[38] Boyle T Connors JM Gascoyne RD Berry BR Sehn LH Bashash M . Physical activity, obesity and survival in diffuse large B-cell and follicular lymphoma cases. Br J Haematol. (2017) 178:442–7. doi: 10.1111/bjh.14702 28466570

[39] Bendtsen MD Munksgaard PS Severinsen MT Bekric E Brieghel C Nielsen KB . Anthropometrics and prognosis in diffuse large B-cell lymphoma: a multicentre study of 653 patients. Eur J Haematol. (2017) 98:355–62. doi: 10.1111/ejh.12835 27893172

[40] Besutti G Massaro F Bonelli E Braglia L Casali M Versari A . Prognostic impact of muscle quantity and quality and fat distribution in diffuse large b-cell lymphoma patients. Front Nutr. (2021) 8:620696. doi: 10.3389/fnut.2021.620696 34026803 PMC8138563

[41] Coutinho R Lobato J Esteves S Cabeçadas J Gomes da. Silva M. Clinical risk scores do not accurately identify a very high risk population with diffuse large B cell lymphoma-an analysis of 386 Portuguese patients. Ann Hematol. (2019) 98:1937–46. doi: 10.1007/s00277-019-03676-0 30949752

[42] Mörth C Valachis A Abu Sabaa A Marshall K Hedström G Flogegård M . Autoimmune disease in patients with diffuse large B-cell lymphoma: occurrence and impact on outcome. Acta Oncol. (2019) 58:1170–7. doi: 10.1080/0284186X.2019.1619936 31131659

[43] Sungur S Yao Y Wellard C Chung E Morgan S Lee D . Body mass index and clinical associations in Australasian lymphoma patients: a lymphoma and related diseases registry study. EJHaem. (2026) 7:e70212. doi: 10.1002/jha2.70212

[44] Wang Z Luo S Zhao X. The prognostic impact of body mass index in patients with diffuse large B-cell lymphoma: a meta-analysis. Nutr Cancer. (2021) 73:2336–46. doi: 10.1080/01635581.2020.1823437 32964748

[45] Rodvold KA Rushing DA Tewksbury DA. Doxorubicin clearance in the obese. J Clin Oncol. (1988) 6:1321–7. doi: 10.1200/JCO.1988.6.8.1321 3411343

[46] Hanley MJ Abernethy DR Greenblatt DJ. Effect of obesity on the pharmacokinetics of drugs in humans. Clin Pharmacokinet. (2010) 49:71–87. doi: 10.2165/11318100-000000000-00000 20067334

[47] Jain R Chung SM Jain L Khurana M Lau SW Lee JE . Implications of obesity for drug therapy: limitations and challenges. Clin Pharmacol Ther. (2011) 90:77–89. doi: 10.1038/clpt.2011.104 21633345

[48] Akazawa N Kishi M Hino T Tsuji R Tamura K Hioka A . Higher body mass index in hospitalized older patients is related to higher muscle quality. J Nutr Health Aging. (2022) 26:495–500. doi: 10.1007/s12603-022-1785-9 35587762 PMC12879151

[49] Niiyama-Uchibori Y Okamoto H Miyashita A Mizuhara K Kanayama-Kawaji Y Fujino T . Skeletal muscle index impacts the treatment outcome of elderly patients with diffuse large B cell lymphoma. Hematol Oncol. (2024) 42:e3252. doi: 10.1002/hon.3252 38287527

[50] Hontecillas-Prieto L Garcia-Dominguez DJ Jimenez-Cortegana C Nogales-Fernandez E Palazon-Carrion N Garcia-Sancho AM . Obesity and overweight in R/R DLBCL patients is associated with a better response to treatment of R2-GDP-GOTEL trial. Potential role of NK CD8 + cells and vitamin D. Cancer Metab. (2025) 13:12. doi: 10.1186/s40170-025-00381-7 40038834 PMC11881355

[51] Renehan AG Harvie M Cutress RI Leitzmann M Pischon T Howell S . How to manage the obese patient with cancer. J Clin Oncol. (2016) 34:4284–94. doi: 10.1200/JCO.2016.69.1899 27903151

[52] Baracos VE Arribas L. Sarcopenic obesity: hidden muscle wasting and its impact for survival and complications of cancer therapy. Ann Oncol. (2018) 29:ii1–9. doi: 10.1093/annonc/mdx810 29506228

[53] Samimi A Ghanavat M Shahrabi S Azizidoost S Saki N. Role of bone marrow adipocytes in leukemia and chemotherapy challenges. Cell Mol Life Sci. (2019) 76:2489–97. doi: 10.1007/s00018-019-03031-6 30715556 PMC11105633

[54] Pramanik R Sheng X Ichihara B Heisterkamp N Mittelman SD. Adipose tissue attracts and protects acute lymphoblastic leukemia cells from chemotherapy. Leuk Res. (2013) 37:503–9. doi: 10.1016/j.leukres.2012.12.013 23332453 PMC3622767

[55] Weihrauch-Blüher S Schwarz P Klusmann JH. Childhood obesity: increased risk for cardiometabolic disease and cancer in adulthood. Metabolism. (2019) 92:147–52. doi: 10.1016/j.metabol.2018.12.001 30529454

[56] Bendor CD Bardugo A Pinhas-Hamiel O Afek A Twig G. Cardiovascular morbidity, diabetes and cancer risk among children and adolescents with severe obesity. Cardiovasc Diabetol. (2020) 19:79. doi: 10.1186/s12933-020-01052-1 32534575 PMC7293793

[57] Vázquez-Vela ME Torres N Tovar AR. White adipose tissue as endocrine organ and its role in obesity. Arch Med Res. (2008) 39:715–28. doi: 10.1016/j.arcmed.2008.09.005 18996284

[58] Galic S Oakhill JS Steinberg GR. Adipose tissue as an endocrine organ. Mol Cell Endocrinol. (2010) 316:129–39. doi: 10.1016/j.mce.2009.08.018 19723556

[59] Gilbert CA Slingerland JM. Cytokines, obesity, and cancer: new insights on mechanisms linking obesity to cancer risk and progression. Annu Rev Med. (2013) 64:45–57. doi: 10.1146/annurev-med-121211-091527 23121183

[60] Tilg H Moschen AR. Adipocytokines: mediators linking adipose tissue, inflammation and immunity. Nat Rev Immunol. (2006) 6:772–83. doi: 10.1038/nri1937 16998510

[61] Turbitt WJ Collins SD Meng H Rogers CJ. Increased adiposity enhances the accumulation of mdscs in the tumor microenvironment and adipose tissue of pancreatic tumor-bearing mice and in immune organs of tumor-free hosts. Nutrients. (2019) 11:3012. doi: 10.3390/nu11123012 31835454 PMC6950402

[62] Han S Jeong AL Lee S Park JS Kim KD Choi I . Adiponectin deficiency suppresses lymphoma growth in mice by modulating NK cells, CD8 T cells, and myeloid-derived suppressor cells. J Immunol. (2013) 190:4877–86. doi: 10.4049/jimmunol.1202487 23530146

[63] Kim KY Kim JK Han SH Lim JS Kim KI Cho DH . Adiponectin is a negative regulator of NK cell cytotoxicity. J Immunol. (2006) 176:5958–64. doi: 10.4049/jimmunol.176.10.5958 16670304

[64] Gruberg L Weissman NJ Waksman R Fuchs S Deible R Pinnow EE . The impact of obesity on the short-term and long-term outcomes after percutaneous coronary intervention: the obesity paradox? J Am Coll Cardiol. (2002) 39:578–84. doi: 10.1016/S0735-1097(01)01802-2 11849854

[65] Bataillard EJ Cheah CY Maurer MJ Khurana A Eyre TA El-Galaly TC. Impact of R-CHOP dose intensity on survival outcomes in diffuse large B-cell lymphoma: a systematic review. Blood Adv. (2021) 5:2426–37. doi: 10.1182/bloodadvances.2021004665 33961018 PMC8114545

[66] Długosz-Danecka M Szmit S Ogórka T Skotnicki AB Jurczak W. The average relative dose intensity of R-CHOP is an independent factor determining favorable overall survival in diffuse large B-cell lymphoma patients. Cancer Med. (2019) 8:1103–9. doi: 10.1002/cam4.2008 30740919 PMC6434223

[67] Lee S Fujita K Negoro E Morishita T Oiwa K Tsukasaki H . Impact of relative dose intensity of standard regimens on survival in elderly patients aged 80 years and older with diffuse large B-cell lymphoma. Haematologica. (2020) 105:e415–8. doi: 10.3324/haematol.2019.234435 31919079 PMC7395278

[68] Wudhikarn K Bansal R Khurana A Hathcock MA Bennani NN Paludo J . The impact of obesity and body weight on the outcome of patients with relapsed/refractory large B-cell lymphoma treated with axicabtagene ciloleucel. Blood Cancer J. (2021) 11:124. doi: 10.1038/s41408-021-00515-2 34210955 PMC8249448

[69] Griggs JJ Bohlke K Balaban EP Dignam JJ Hall ET Harvey RD . Appropriate systemic therapy dosing for obese adult patients with cancer: ASCO guideline update. J Clin Oncol. (2021) 39:2037–48. doi: 10.1200/JCO.21.00471 33939491

[70] Bhimani J O'Connell K Gallagher GB Blinder VS Burganowski R Ergas IJ . Understanding the association between body mass index and chemotherapy dose reductions in women treated for stage I-IIIA breast cancer. NPJ Breast Cancer. (2025) 11:119. doi: 10.1038/s41523-025-00833-9 41162408 PMC12572154

[71] Poletto S Novo M Paruzzo L Frascione PMM Vitolo U. Treatment strategies for patients with diffuse large B-cell lymphoma. Cancer Treat Rev. (2022) 110:102443. doi: 10.1016/j.ctrv.2022.102443 35933930

[72] Bosly A Bron D Van Hoof A De Bock R Berneman Z Ferrant A . Achievement of optimal average relative dose intensity and correlation with survival in diffuse large B-cell lymphoma patients treated with CHOP. Ann Hematol. (2008) 87:277–83. doi: 10.1007/s00277-007-0399-y 17952688

[73] Berriel Diaz M Rohm M Herzig S. Cancer cachexia: multilevel metabolic dysfunction. Nat Metab. (2024) 6:2222–45. doi: 10.1038/s42255-024-01167-9 39578650

[74] Porporato PE. Understanding cachexia as a cancer metabolism syndrome. Oncogenesis. (2016) 5:e200. doi: 10.1038/oncsis.2016.3 26900952 PMC5154342

[75] Vaughan VC Martin P Lewandowski PA. Cancer cachexia: impact, mechanisms and emerging treatments. J Cachexia Sarcopenia Muscle. (2013) 4:95–109. doi: 10.1007/s13539-012-0087-1 23097000 PMC3684701

[76] Takaoka T Yaegashi A Watanabe D. Prevalence of and survival with cachexia among patients with cancer: a systematic review and meta-analysis. Adv Nutr. (2024) 15:100282. doi: 10.1016/j.advnut.2024.100282 39127425 PMC11402144

[77] Lena A Wilkenshoff U Hadzibegovic S Porthun J Rösnick L Fröhlich AK . Clinical and prognostic relevance of cardiac wasting in patients with advanced cancer. J Am Coll Cardiol. (2023) 81:1569–86. doi: 10.1016/j.jacc.2023.02.039 37076211

[78] Anker MS Sanz AP Zamorano JL Mehra MR Butler J Riess H . Advanced cancer is also a heart failure syndrome: a hypothesis. Eur J Heart Fail. (2021) 23:140–4. doi: 10.1002/ejhf.2071 33247608

[79] Tian M Nishijima Y Asp ML Stout MB Reiser PJ Belury MA. Cardiac alterations in cancer-induced cachexia in mice. Int J Oncol. (2010) 37:347–53. doi: 10.3892/ijo_00000683 20596662

