# Supplementary material for: Evaluating the prognostic value of body mass index in diffuse large B-cell lymphoma: a systematic review and meta-analysis
Source: Front Nutr. 2026 Jun 23;13:1762123. doi: 10.3389/fnut.2026.1762123 (PMC13337460; doi:10.3389/fnut.2026.1762123)
Supplement: Supplementary file 1 [file Table_1.docx]

**Evaluating the Prognostic Value of Body Mass Index in Diffuse Large B-Cell Lymphoma: A Systematic Review and Meta-Analysis**

**“Online Supplementary Material”**

**Supplemental Appendix 1:**

Full search strategy (14th April, 2026): PubMed

| **Searches** |
| --- |
| #1 ("Lymphoma, Large B-Cell, Diffuse"[Mesh]) OR (((((Diffuse Large B-Cell Lymphoma[Title/Abstract]) OR (Diffuse Large B Cell Lymphoma[Title/Abstract])) OR (Lymphoma, Large Lymphoid, Diffuse[Title/Abstract])) OR (Diffuse, Large B-Cell, Lymphoma[Title/Abstract])) OR (DLBCL[Title/Abstract]))  #2 ("Body Mass Index"[MeSH Terms]) OR ("Body Mass Index"[Title/Abstract] OR "BMI"[Title/Abstract] OR "body weight"[Title/Abstract] OR "body mass"[Title/Abstract] OR "body size"[Title/Abstract] OR "body fat"[Title/Abstract] OR "body composition"[Title/Abstract] OR "adiposity"[Title/Abstract] OR "obesity"[Title/Abstract] OR "obese"[Title/Abstract] OR "overweight"[Title/Abstract] OR "underweight"[Title/Abstract])  #3 #1 AND #2  #4 "animals"[MeSH Terms] NOT "humans"[MeSH Terms]  #5 #3 NOT #4  #6 limit #5 to English language |

**Supplemental Appendix 2:** Full-text publications excluded with reason

| **Online Supplementary Reference** | **Reason for exclusion** |
| --- | --- |
| (1) | Inappropriate study type (n=1) |
| (2-4) | Overlapping population (n=3) |
| (5-35) | Inappropriate outcome (n=31) |

**Reference**

1. Chen Y, Zheng T, Lan Q, Kim C, Qin Q, Foss F, et al. Polymorphisms in DNA repair pathway genes, body mass index, and risk of non-Hodgkin lymphoma. American journal of hematology. 2013;88(7):606-11.

2. Go SI, Kim HG, Kang MH, Park S, Lee GW. Prognostic model based on the geriatric nutritional risk index and sarcopenia in patients with diffuse large B-cell lymphoma. BMC Cancer. 2020;20(1):439.

3. Leo QJ, Ollberding NJ, Wilkens LR, Kolonel LN, Henderson BE, Le Marchand L, et al. Obesity and non-Hodgkin lymphoma survival in an ethnically diverse population: the Multiethnic Cohort study. Cancer causes & control : CCC. 2014;25(11):1449-59.

4. Camus V, Lanic H, Kraut J, Modzelewski R, Clatot F, Picquenot JM, et al. Prognostic impact of fat tissue loss and cachexia assessed by computed tomography scan in elderly patients with diffuse large B-cell lymphoma treated with immunochemotherapy. Eur J Haematol. 2014;93(1):9-18.

5. Ariestine DA, Sari NK, Rinaldi I, Abdullah M. Quality of life in older survivors of non-Hodgkin's lymphoma who received chemotherapy and related factors. Journal of Geriatric Oncology. 2021;12(2):326-31.

6. Armenian SH, Iukuridze A, Teh JB, Mascarenhas K, Herrera A, McCune JS, et al. Abnormal body composition is a predictor of adverse outcomes after autologous haematopoietic cell transplantation. Journal of cachexia, sarcopenia and muscle. 2020;11(4):962-72.

7. Atak S, Serin S, Demirel N, Dogan EE, Aydın D, Nizam N, et al. The Obesity Controversy: Does It Impact Treatment Response in Diffuse Large B-Cell Lymphoma? Int J Hematol Oncol Stem Cell Res. 2023;17(2):75-80.

8. Bachanova V, Rogosheske J, Shanley R, Burns LJ, Smith SM, Weisdorf DJ, et al. Adjusting Cyclophosphamide Dose in Obese Patients with Lymphoma Is Safe and Yields Favorable Outcomes after Autologous Hematopoietic Cell Transplantation. Biology of blood and marrow transplantation : journal of the American Society for Blood and Marrow Transplantation. 2016;22(3):571-4.

9. Chen Y, Chen Z, Tan X, Zhang Q, Zhou Y, Yuan H, et al. Role of body composition and metabolic parameters extracted from baseline (18)F-FDG PET/CT in patients with diffuse large B-cell lymphoma. Annals of hematology. 2023;102(10):2779-89.

10. Go SI, Park MJ, Song HN, Kim HG, Kang MH, Kang JH, et al. A comparison of pectoralis versus lumbar skeletal muscle indices for defining sarcopenia in diffuse large B-cell lymphoma - two are better than one. Oncotarget. 2017;8(29):47007-19.

11. Guo J, Cai P, Li P, Cao C, Zhou J, Dong L, et al. Body Composition as a Predictor of Toxicity and Prognosis in Patients with Diffuse Large B-Cell Lymphoma Receiving R-CHOP Immunochemotherapy. Current oncology (Toronto, Ont). 2021;28(2):1325-37.

12. Jaswal S, Sanders V, Pullarkat P, Teja S, Salter A, Watkins MP, et al. Metabolic Biomarkers Assessed with PET/CT Predict Sex-Specific Longitudinal Outcomes in Patients with Diffuse Large B-Cell Lymphoma. Cancers (Basel). 2022;14(12).

13. Niiyama-Uchibori Y, Okamoto H, Miyashita A, Mizuhara K, Kanayama-Kawaji Y, Fujino T, et al. Skeletal muscle index impacts the treatment outcome of elderly patients with diffuse large B cell lymphoma. Hematological oncology. 2024;42(1).

14. O'Brian K, Luo S, Ganti A, Riedell P, Lynch RC, Roop R, et al. Short- and Long-term weight changes among United States veterans with diffuse large B-cell lymphoma treated with CHOP chemotherapy. Leukemia & lymphoma. 2016;57(2):313-9.

15. Okello CD, Omoding A, Ddungu H, Mulumba Y, Orem J. Outcomes of treatment with CHOP and EPOCH in patients with HIV associated NHL in a low resource setting. BMC Cancer. 2020;20(1):798.

16. Park S, Han B, Cho JW, Woo SY, Kim S, Kim SJ, et al. Effect of nutritional status on survival outcome of diffuse large B-cell lymphoma patients treated with rituximab-CHOP. Nutr Cancer. 2014;66(2):225-33.

17. Rier HN, Kharagjitsing H, van Rosmalen J, van Vugt J, Westerweel PE, de Jongh E, et al. Prognostic impact of low muscle mass and low muscle density in patients with diffuse large B-cell lymphoma. Leukemia & lymphoma. 2020;61(7):1618-26.

18. Sosa-Romero JT, Navarrete-Reyes AP, Castillo-Martínez L, Gabutti-Thomas JA, del Pilar Milke-García M, Agreda-Vásquez GP. Sarcopenia in older patients with diffuse large B-cell lymphoma and its association with response to treatment: A cohort study. Journal of Geriatric Oncology. 2023;14(3).

19. Sumransub N, Cao Q, Juckett M, Betts B, Holtan S, Jurdi NE, et al. Sarcopenia Predicts Inferior Progression-Free Survival in Lymphoma Patients Treated with Autologous Hematopoietic Stem Cell Transplantation. Transplantation and Cellular Therapy. 2023;29(4):263.e1-.e7.

20. Wudhikarn K, Bansal R, Khurana A, Hathcock MA, Bennani NN, Paludo J, et al. The impact of obesity and body weight on the outcome of patients with relapsed/refractory large B-cell lymphoma treated with axicabtagene ciloleucel. Blood cancer journal. 2021;11(7):124.

21. Xiao DY, Luo S, O'Brian K, Ganti A, Riedell P, Sanfilippo KM, et al. Impact of sarcopenia on treatment tolerance in United States veterans with diffuse large B-cell lymphoma treated with CHOP-based chemotherapy. American journal of hematology. 2016;91(10):1002-7.

22. Xiao DY, Luo S, O'Brian K, Sanfilippo KM, Ganti A, Riedell P, et al. Longitudinal Body Composition Changes in Diffuse Large B-cell Lymphoma Survivors: A Retrospective Cohort Study of United States Veterans. Journal of the National Cancer Institute. 2016;108(11).

23. Zhou M, Cheng J, Zhao H, Yang M, Yu W, Qin J, et al. Clinical Features, Phenotypic Markers and Outcomes of Diffuse Large B-Cell Lymphoma between HIV-Infected and HIV-Uninfected Chinese Patients. Cancers (Basel). 2022;14(21).

24. Chan H, Jackson S, McLay J, Knox A, Lee J, Wang S, et al. Obese non-Hodgkin lymphoma patients tolerate full uncapped doses of chemotherapy with no increase in toxicity, and a similar survival to that seen in nonobese patients. Leukemia & lymphoma. 2016;57(11):2584-92.

25. Go SI, Park MJ, Park S, Kang MH, Kim HG, Kang JH, et al. Cachexia index as a potential biomarker for cancer cachexia and a prognostic indicator in diffuse large B-cell lymphoma. Journal of cachexia, sarcopenia and muscle. 2021;12(6):2211-9.

26. Kanemasa Y, Shimoyama T, Sasaki Y, Tamura M, Sawada T, Omuro Y, et al. Analysis of the prognostic value of BMI and the difference in its impact according to age and sex in DLBCL patients. Hematological oncology. 2018;36(1):76-83.

27. Simard JF, Baecklund F, Chang ET, Baecklund E, Hjalgrim H, Olov Adami H, et al. Lifestyle factors, autoimmune disease and family history in prognosis of non-hodgkin lymphoma overall and subtypes. Int J Cancer. 2013;132(11):2659-66.

28. Wu YY, You JY, Huang CE, Hsu CC, Chen YY, Tsou HY, et al. Clinicopathological characteristics and treatment outcome in obese patients with diffuse large B-cell lymphoma. Translational cancer research. 2020;9(10):6116-27.

29. Zhou W, Li W, He C, Ma R, Gao Q, Wang Y, et al. Influence of Hyperglycemia on the Prognosis of Patients with Diffuse Large B-Cell Lymphoma. Diabetes, metabolic syndrome and obesity : targets and therapy. 2022;15:2039-49.

30. Du M, Mayombo RTM, Liu J, Zhang Y, Liao D, Hu Y, et al. The impact of obesity and its related underlying diseases on cytokine release syndrome and the efficacy of CAR-T therapy in treating B-cell malignancies. Annals of hematology. 2025;104(3):1887-95.

31. Fujita K, Tsukasaki H, Lee S, Morishita T, Negoro E, Oiwa K, et al. Age interaction in associations between geriatric nutritional risk index and prognosis in diffuse large B-cell lymphoma. Annals of hematology. 2025;104(11):5819-28.

32. Hontecillas-Prieto L, García-Domínguez DJ, Jiménez-Cortegana C, Nogales-Fernández E, Palazón-Carrión N, García-Sancho AM, et al. Obesity and overweight in R/R DLBCL patients is associated with a better response to treatment of R2-GDP-GOTEL trial. Potential role of NK CD8 + cells and vitamin D. Cancer Metab. 2025;13(1):12.

33. Hontecillas-Prieto L, García-Domínguez DJ, Jiménez-Cortegana C, Nogales-Fernández E, Palazón-Carrión N, García-Sancho AM, et al. High leptin levels independent of body mass index are associated with inflammation and poorer treatment response in patients with diffuse large B-cell lymphoma. Biomedicine & pharmacotherapy = Biomedecine & pharmacotherapie. 2026;195:118975.

34. Tan JY, Yeo YH, Wong HKK, Elemian S, Mir M, Ang QX, et al. Impact of Obesity on Patients with Diffuse Large B-Cell Lymphoma Receiving Chimeric Antigen Receptor T-Cell Therapy. Oncology. 2026;104(4):463-7.

35. Zeng C, Wei Z, Huang J, Zhu J, Sun F, Wang J, et al. Effect of body mass index on the prognosis of children and adolescents with high-grade mature B-cell non-Hodgkin lymphoma. Cancer. 2024;130(23):4109-17.

**Supplemental Table 1.** Study-specific BMI classification criteria used in the included studies

| Study | Year | Location | Underweight | Normal-weight | Overweight | Obesity |
| --- | --- | --- | --- | --- | --- | --- |
| Bendtsen et al. | 2017 | Denmark | <18.5 kg/m^2^ | 18.5–24.9 kg/m^2^ | 25.0–29.9 kg/m^2^ | ≥30.0 kg/m^2^ |
| Besutti et al. | 2021 | Italy | NR | NR | NR | ≥30.0 kg/m^2^ |
| Boyle et al. | 2017 | Canada | NR | <25.0 kg/m^2^ | 25.0–29.9 kg/m^2^ | ≥30.0 kg/m^2^ |
| Carson et al. | 2012 | USA | <18.5 kg/m^2^ | 18.5 to <25.0 kg/m^2^ | 25.0 to <30.0 kg/m^2^ | ≥30.0 kg/m^2^ |
| Chihara et al. | 2021 | USA | <18.5 kg/m^2^ | 18.5–24.9 kg/m^2^ | 25.0–29.9 kg/m^2^ | ≥30.0 kg/m^2^ |
| Coutinho et al. | 2019 | Portugal | <25 kg/m^2^ | | ＞24.9 kg/m^2^ | |
| Detroit et al. | 2023 | France | <25 kg/m^2^ | | ＞24.9 kg/m^2^ | |
| Geyer et al. | 2010 | USA | <20.0 kg/m^2^ | 20.0–24.9 kg/m^2^ | 25.0–29.9 kg/m^2^ | ≥30.0 kg/m^2^ |
| Go et al.* | 2019 | Korea | ≤23.0 kg/m^2^ | | ≥23.0 kg/m^2^ | |
| Hong et al. | 2014 | USA | <18.5 kg/m^2^ | 18.5 to <25.0 kg/m^2^ | 25.0 to <30.0 kg/m^2^ | ≥30.0 kg/m^2^ |
| Jones et al. | 2010 | USA | <18.5 kg/m^2^ | 18.5 to <25.0 kg/m^2^ | 25.0 to <30.0 kg/m^2^ | ≥30.0 kg/m^2^ |
| Lanic et al. | 2014 | France | <25 kg/m^2^ | | ≥25 kg/m^2^ | |
| Li et al.* | 2016 | China | ≤23.0 kg/m^2^ | | ≥23.0 kg/m^2^ | |
| Mörth et al. | 2019 | Sweden | <25 kg/m^2^ | | ≥25 kg/m^2^ | |
| Pénichoux et al. | 2023 | France | <25.0 kg/m^2^ | | 25.0 to <30.0 kg/m^2^ | ≥30.0 kg/m^2^ |
| Sarkozy et al. | 2014 | France | <18.5 kg/m^2^ | 18.5–25.0 kg/m^2^ | >25.0 kg/m^2^ | >30.0 kg/m^2^ |
| Shin et al.* | 2016 | Korea | <18.5 kg/m^2^ | 18.5–<23.0 kg/m^2^ | 23.0–24.9 kg/m^2^ | ≥25.0 kg/m^2^ |
| Weiss et al. | 2014 | Austria | <18.5 kg/m^2^ | 18.5 to <25.0 kg/m^2^ | 25.0 to <30.0 kg/m^2^ | ≥30.0 kg/m^2^ |
| Yang et al.* | 2021 | China | <23 .0 kg/m^2^ | | ≥23.0 kg/m^2^ | |
| Zhou et al. | 2016 | USA | ≤18.5 kg/m^2^ | >18.5–25.0 kg/m^2^ | >25.0 kg/m^2^ | |
| Penichoux et al. | 2025 | France | <18.5 kg/m^2^ | 18.5 to <25.0 kg/m^2^ | 25.0 to <30.0 kg/m^2^ | >30.0 kg/m^2^ |
| Hwang et al.* | 2015 | Korea | <18.5 kg/m^2^ | 18.5–22.9 kg/m^2^ | 23.0–24.9 kg/m^2^ | >25.0 kg/m^2^ |
| Sungur et al. | 2026 | Australasia | <18.5 kg/m^2^ | 18.5–24.9 kg/m^2^ | 25.0–30.0 kg/m^2^ | >30.0 kg/m^2^ |

NR, not reported. Studies marked with an asterisk used an Asian-specific BMI cutoff.


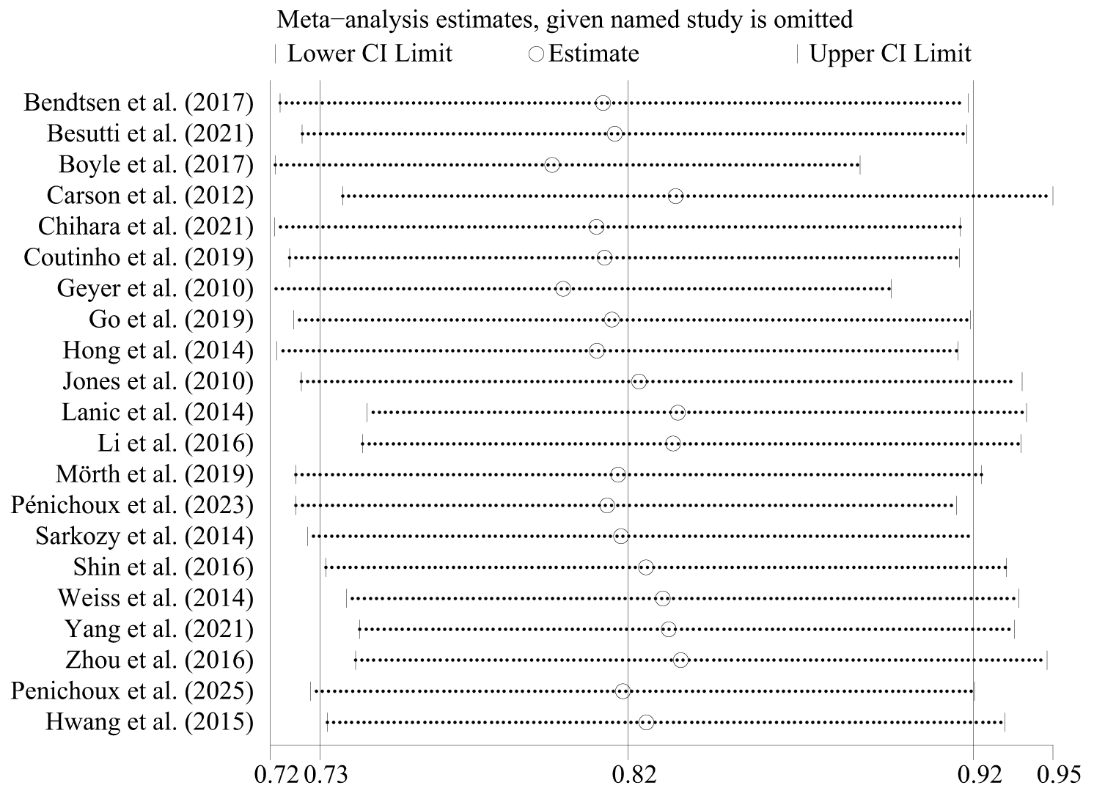


**Supplemental Figure 1:** Sensitivity analysis of the association between overweight/obesity and OS in patients with DLBCL, compared with patients without overweight/obesity. CI: confidence interval.


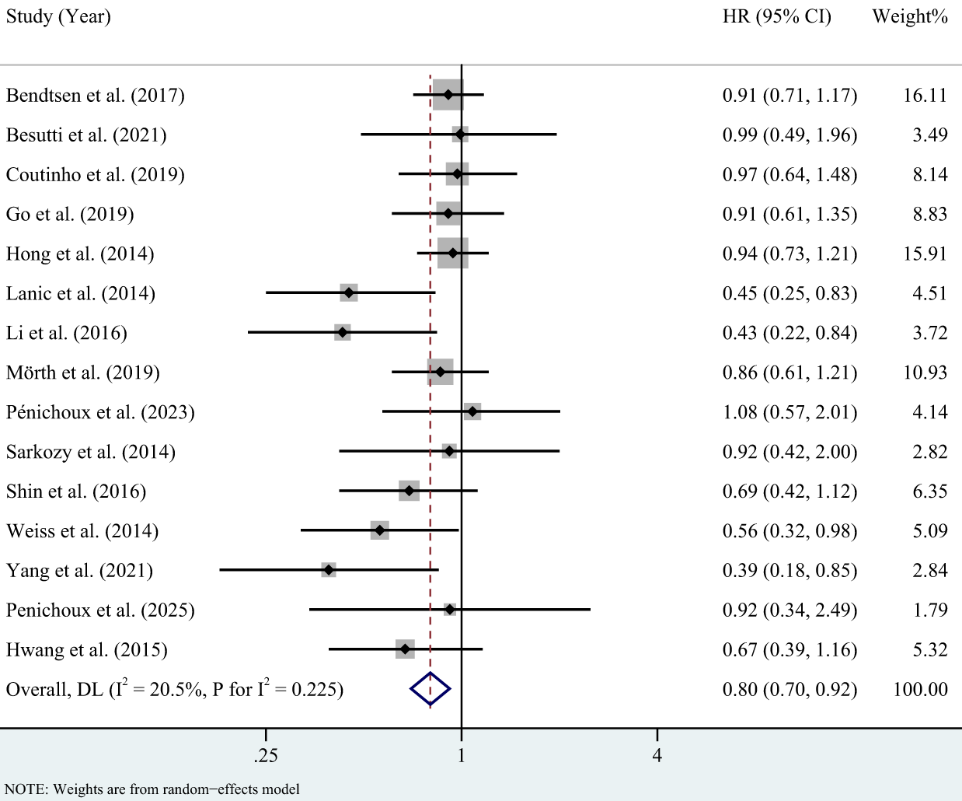


**Supplemental Figure 2:** Forest plot of the subgroup analysis evaluating the association between BMI and OS in patients with DLBCL treated with R-CHOP-like regimens (hazard ratios). CI, confidence interval; DL, DerSimonian-Laird estimate; I^2^, inconsistency; P for I^2^, P value for the test of between-study heterogeneity.


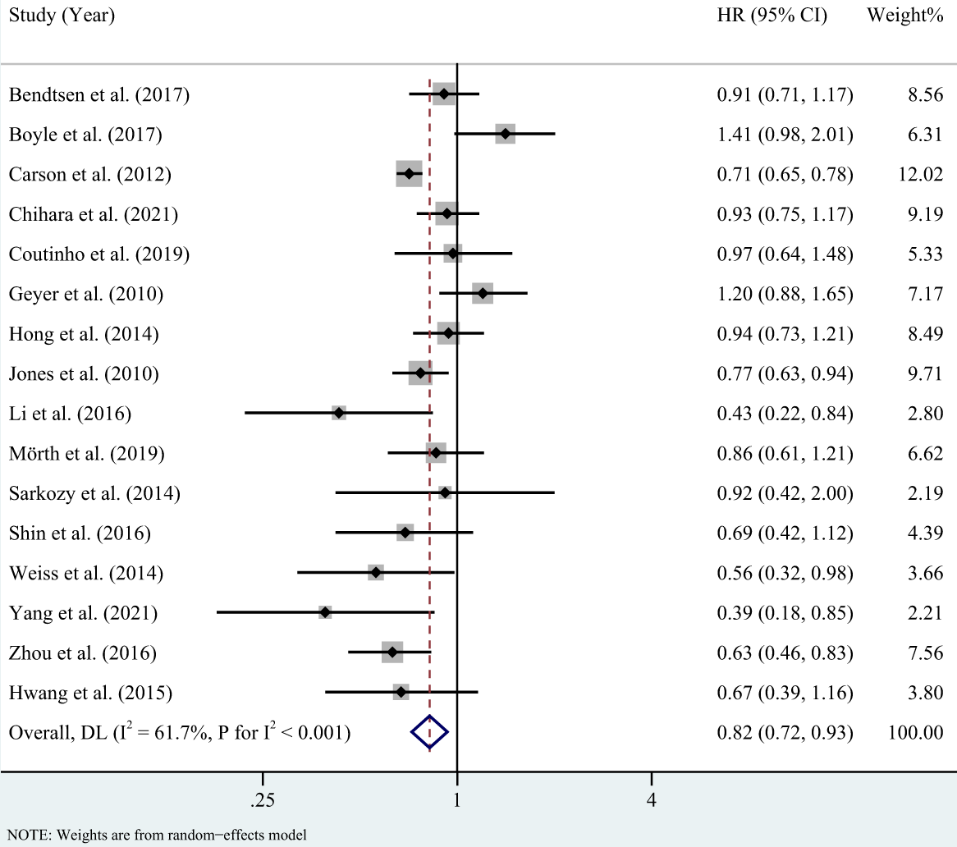


**Supplemental Figure 3:** Forest plot of the subgroup analysis evaluating the association between BMI and OS in patients with DLBCL, restricted to studies reporting multivariable analyses (hazard ratios). CI, confidence interval; DL, DerSimonian-Laird estimate; I^2^, inconsistency; P for I^2^, P value for the test of between-study heterogeneity.


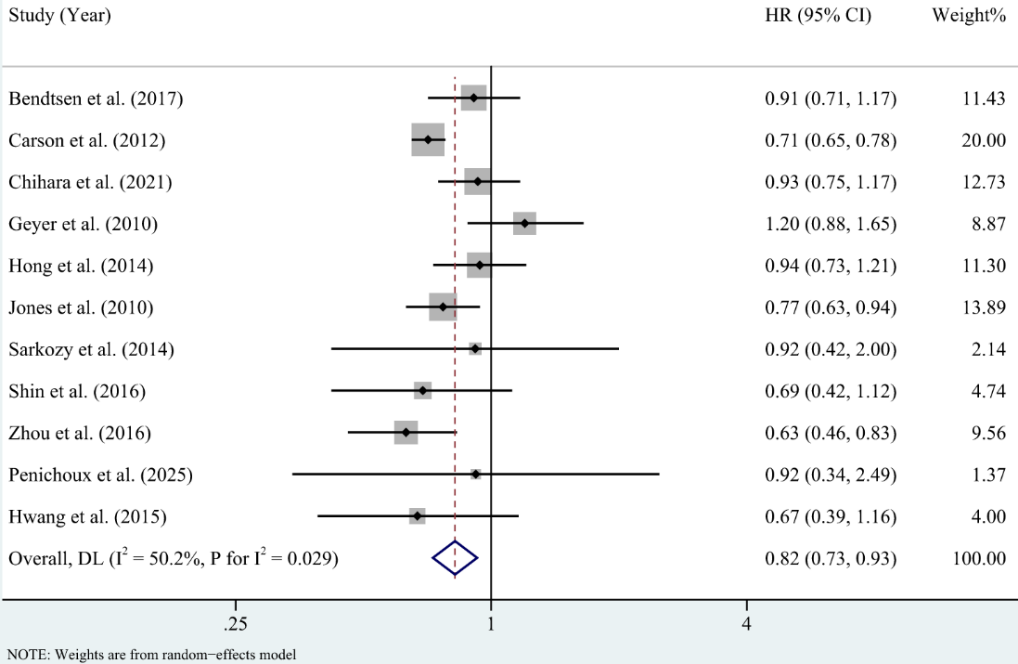


**Supplemental Figure 4:** Forest plot of the subgroup analysis evaluating the association between BMI and OS in patients with DLBCL, with the reference group restricted to normal-weight patients and underweight patients excluded (hazard ratios). CI, confidence interval; DL, DerSimonian-Laird estimate; I^2^, inconsistency; P for I^2^, P value for the test of between-study heterogeneity.


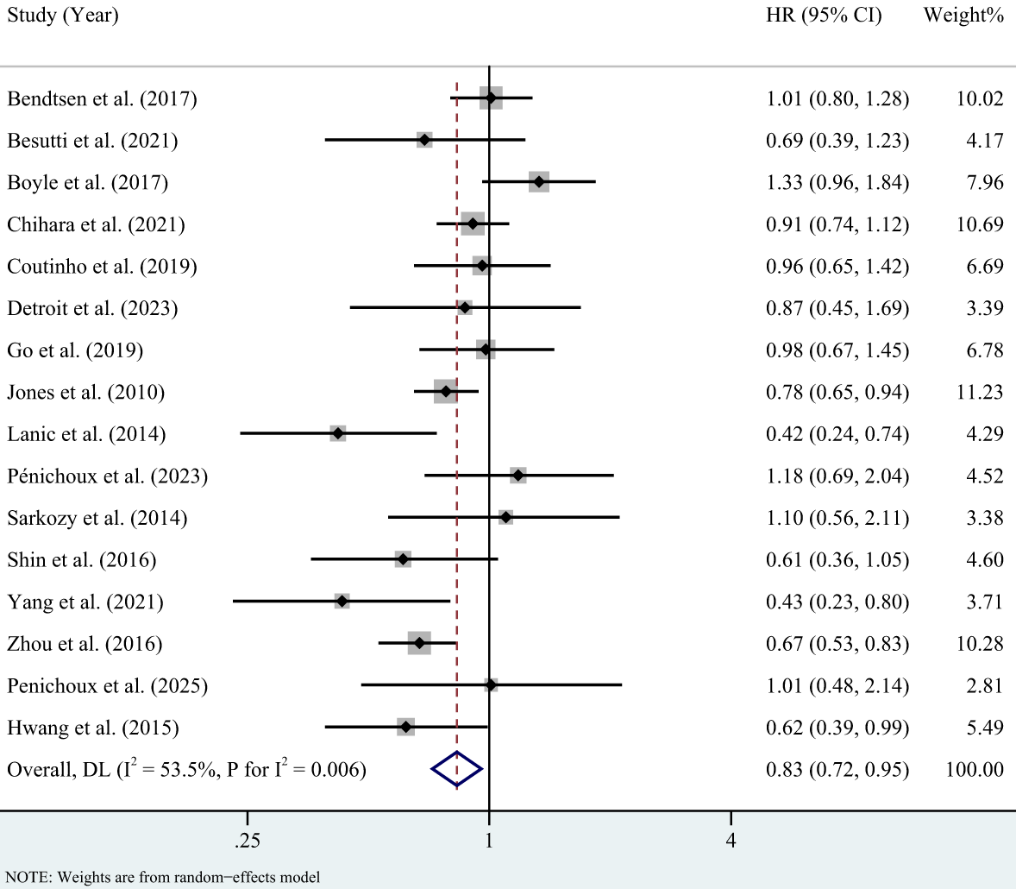


**Supplemental Figure 5:** Forest plot of the subgroup analysis evaluating the association between BMI and PFS in patients with DLBCL, including the study of CAR T-cell therapy (hazard ratios). CI, confidence interval; DL, DerSimonian-Laird estimate; I^2^, inconsistency; P for I^2^, P value for the test of between-study heterogeneity.


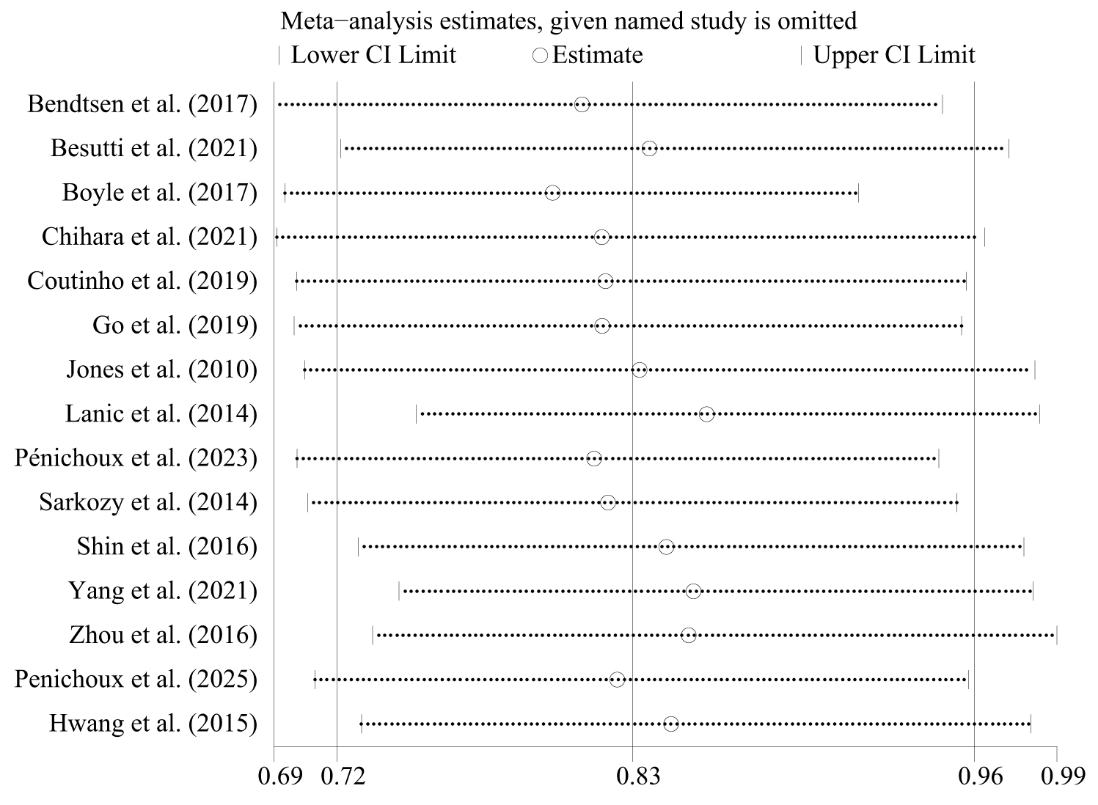


**Supplemental Figure 6:** Sensitivity analysis of the association between overweight/obesity and PFS in patients with DLBCL, compared with patients without overweight/obesity. CI: confidence interval.


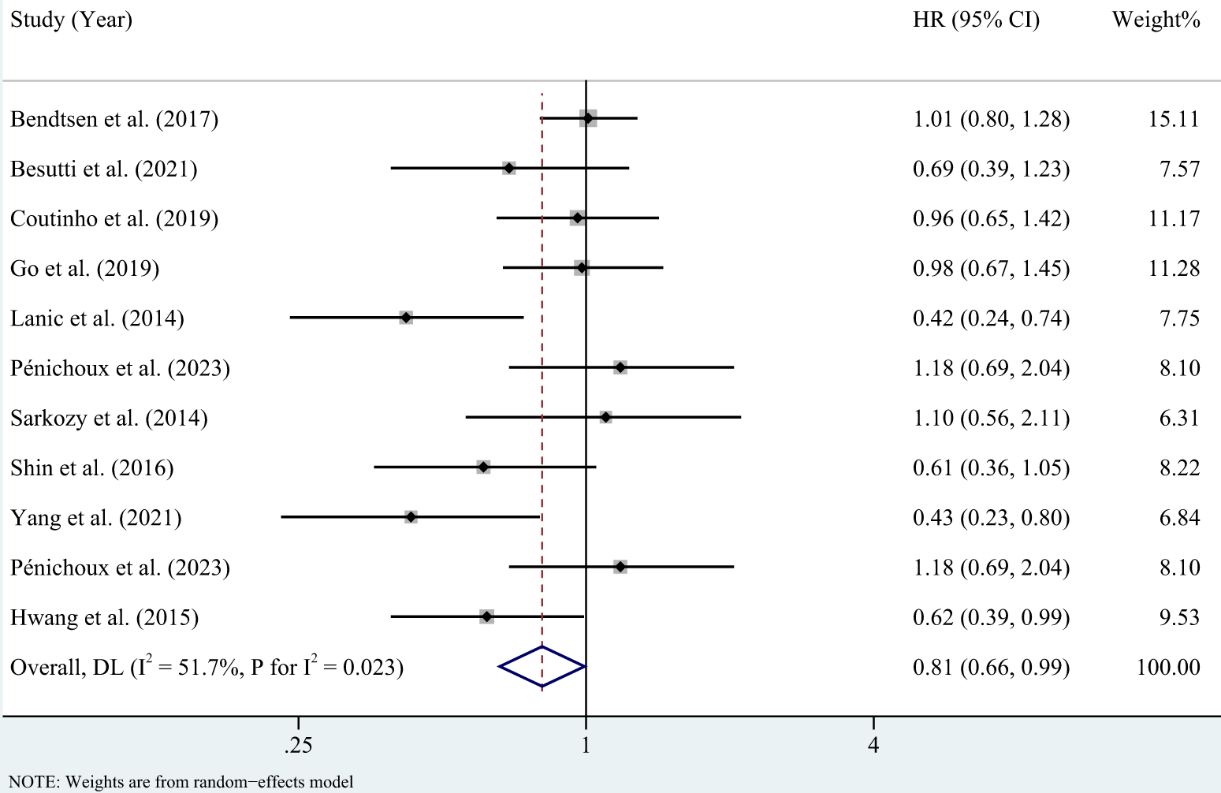


**Supplemental Figure 7:** Forest plot of the subgroup analysis evaluating the association between BMI and PFS in patients with DLBCL treated with R-CHOP-like regimens (hazard ratios). CI, confidence interval; DL, DerSimonian-Laird estimate; I^2^, inconsistency; P for I^2^, P value for the test of between-study heterogeneity.


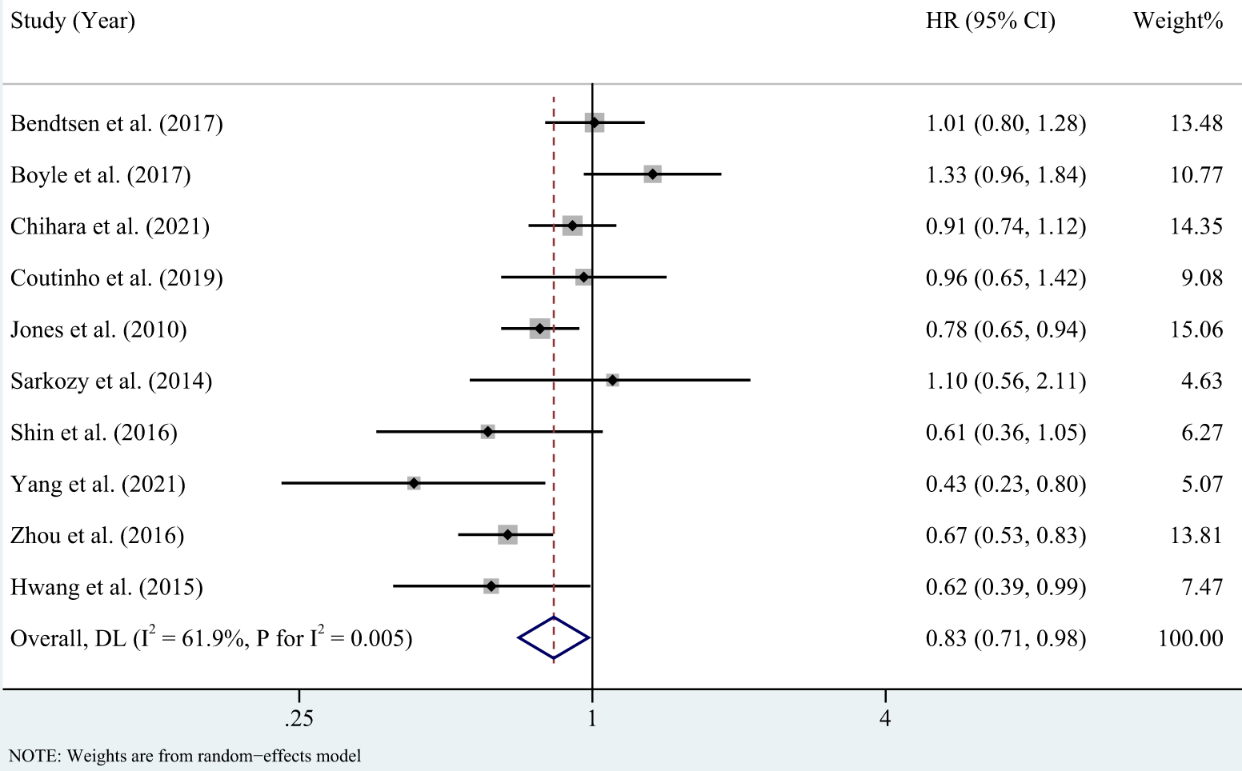


**Supplemental Figure 8:** Forest plot of the subgroup analysis evaluating the association between BMI and PFS in patients with DLBCL, restricted to studies reporting multivariable analyses (hazard ratios). CI, confidence interval; DL, DerSimonian-Laird estimate; I^2^, inconsistency; P for I^2^, P value for the test of between-study heterogeneity.


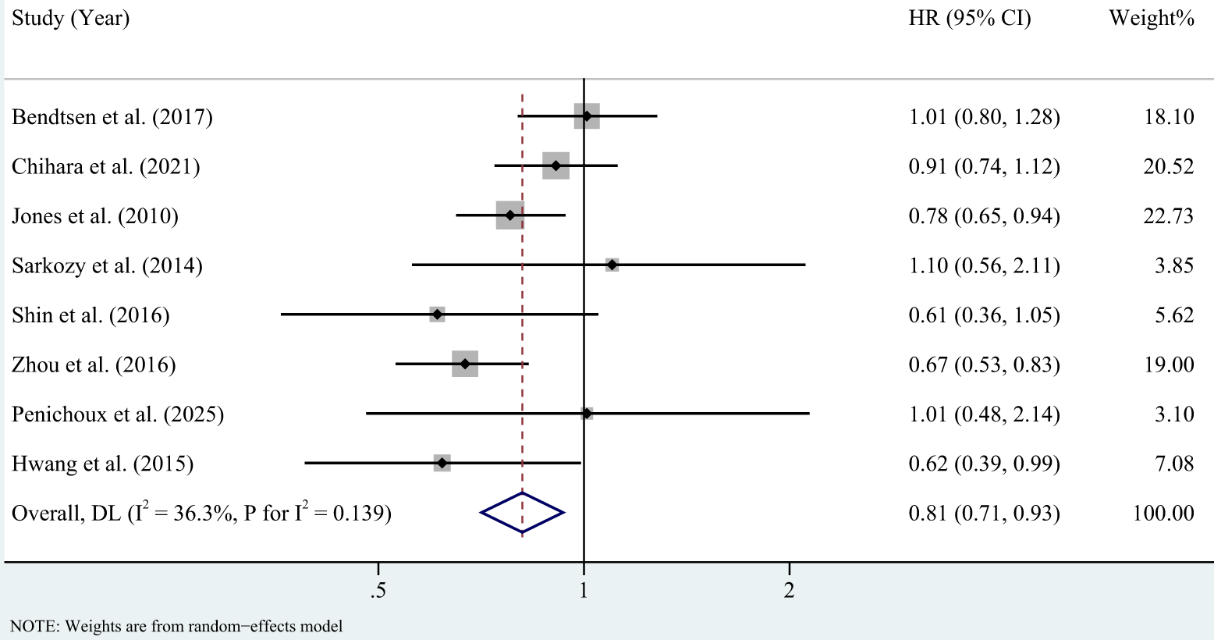


**Supplemental Figure 9:** Forest plot of the subgroup analysis evaluating the association between BMI and PFS in patients with DLBCL, with the reference group restricted to normal-weight patients and underweight patients excluded (hazard ratios). CI, confidence interval; DL, DerSimonian-Laird estimate; I², inconsistency; P for I², P value for the test of between-study heterogeneity.


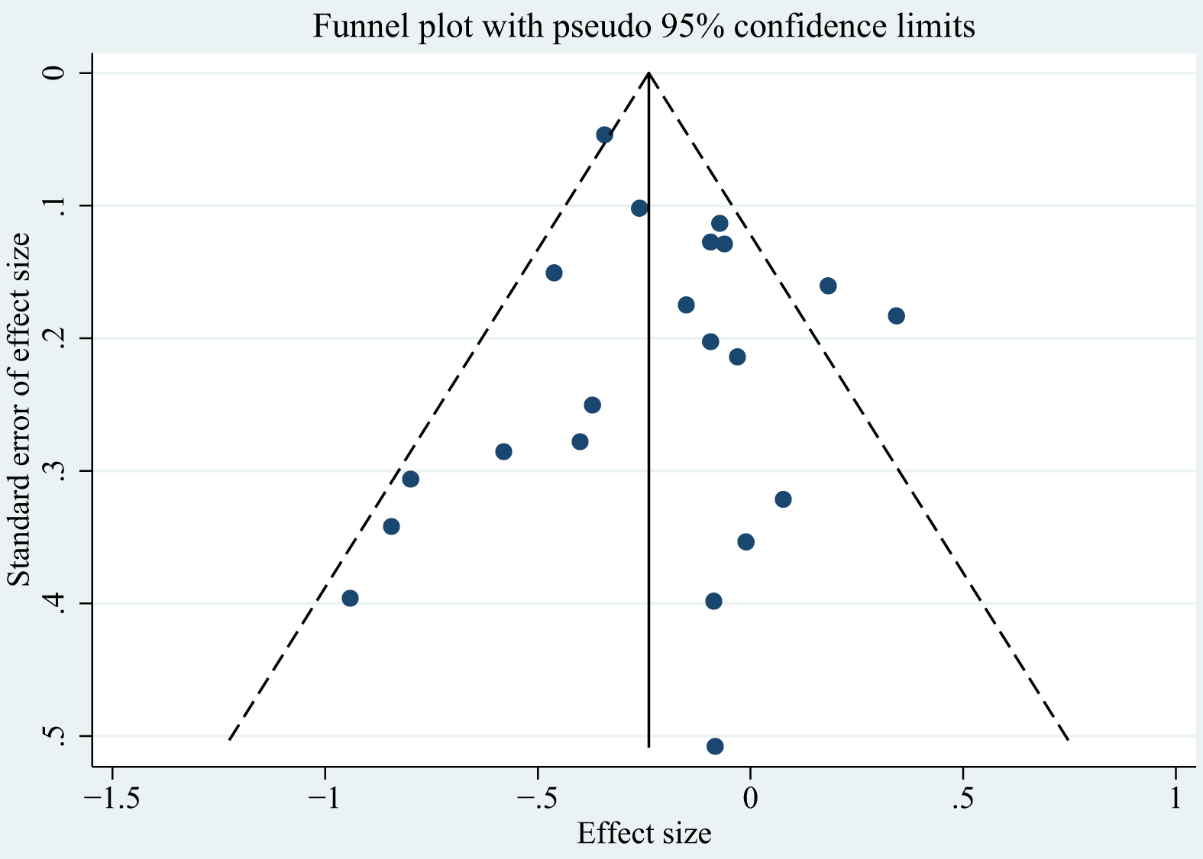


**Supplemental Figure 10**: Funnel plot evaluating the association between BMI and OS.


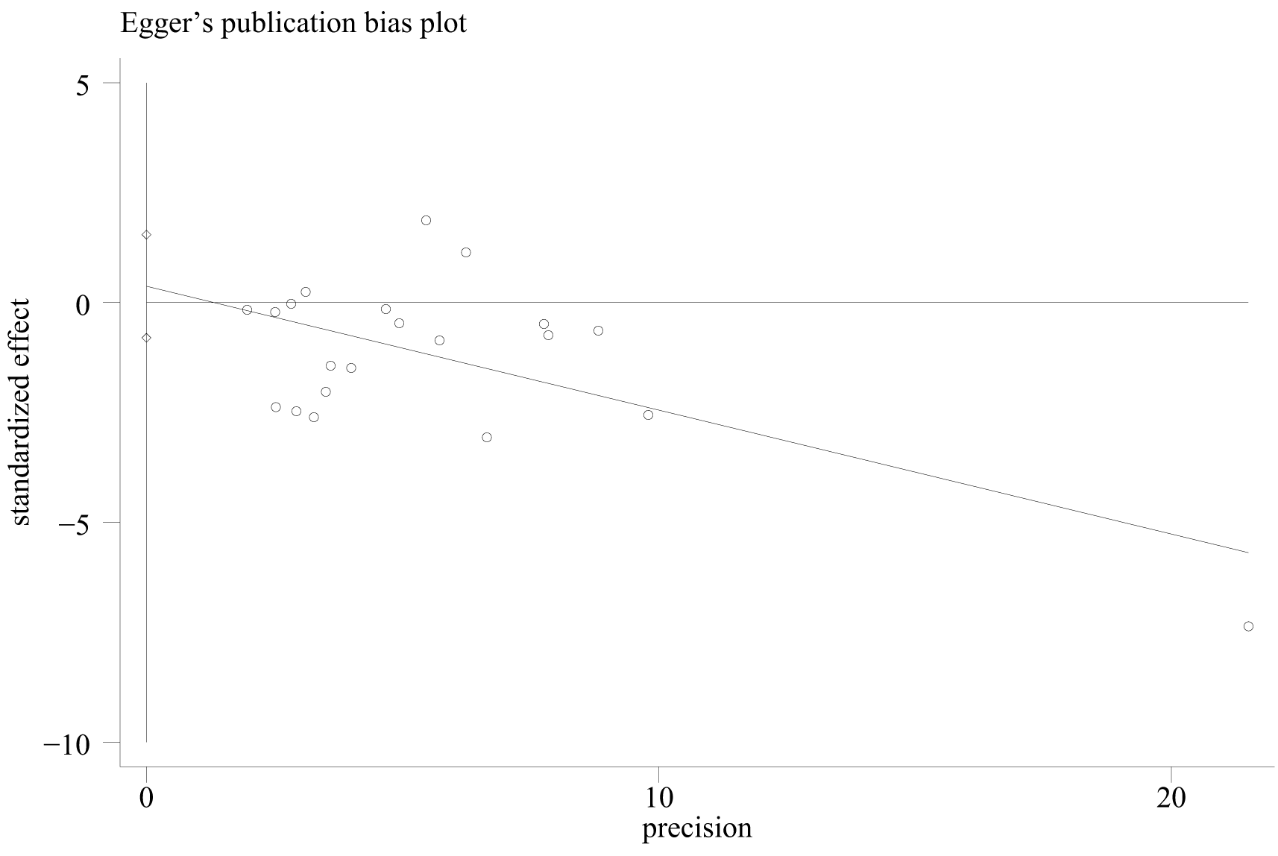


**Supplemental Figure 11**: The Egger’s test of the associations between BMI and OS.


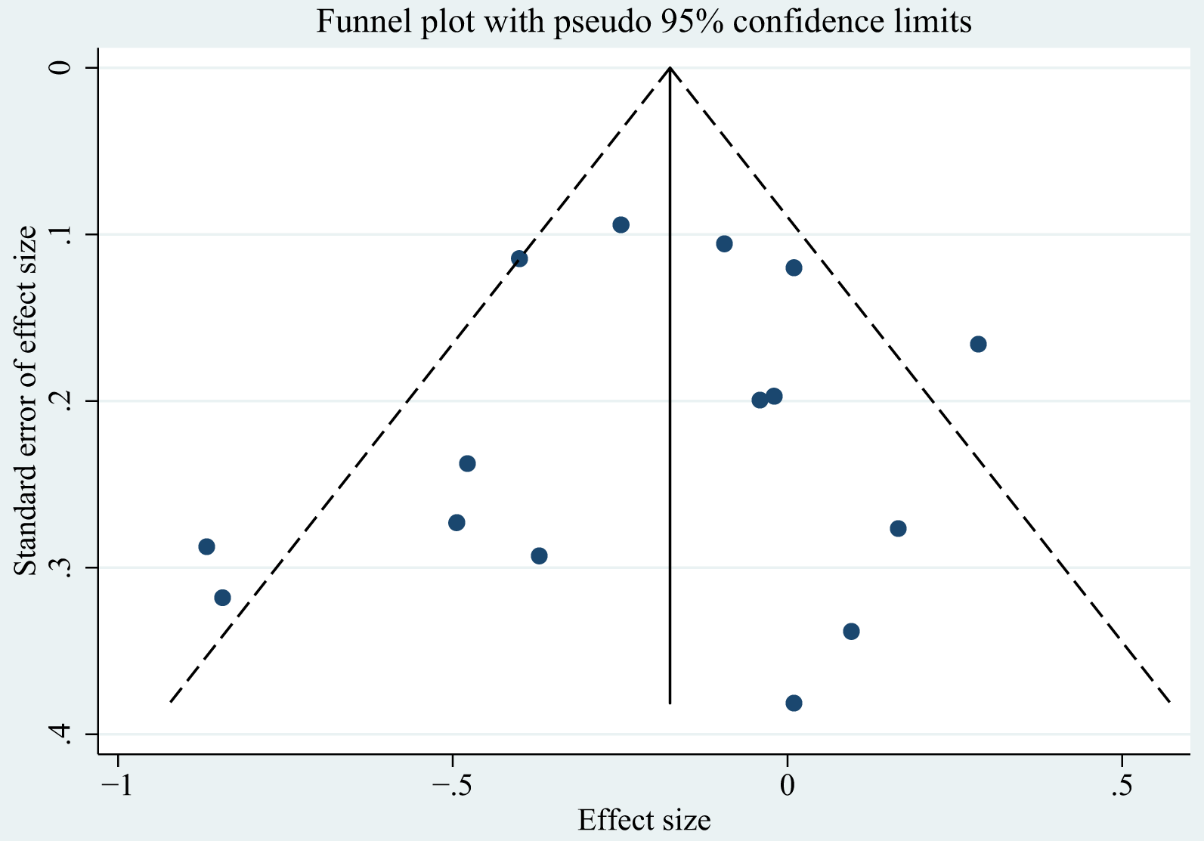


**Supplemental Figure 12**: Funnel plot evaluating the association between BMI and PFS.


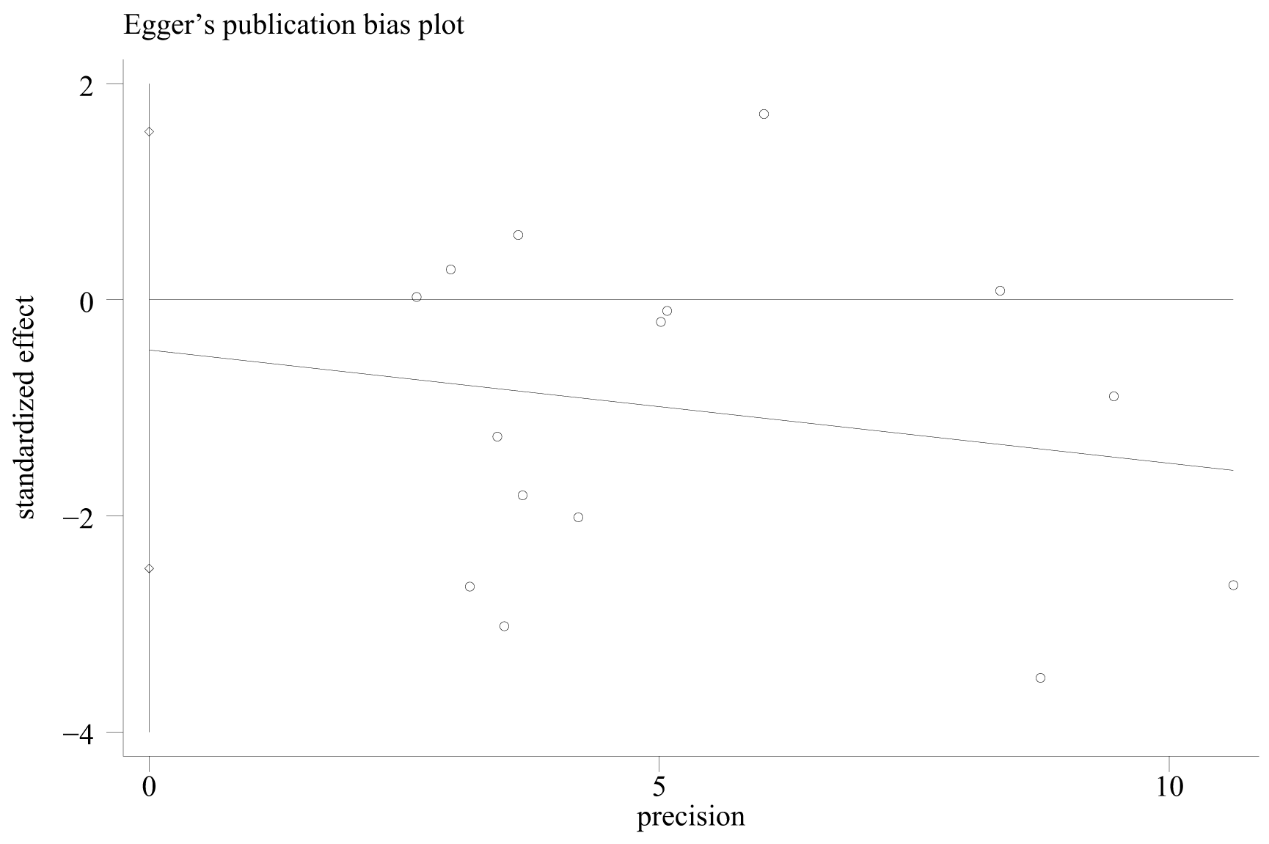


**Supplemental Figure 13**: The Egger’s test of the associations between BMI and PFS.
